# Photothermal and Photodynamic Therapy of Tumors with Plasmonic Nanoparticles: Challenges and Prospects

**DOI:** 10.3390/ma15041606

**Published:** 2022-02-21

**Authors:** Alla B. Bucharskaya, Nikolai G. Khlebtsov, Boris N. Khlebtsov, Galina N. Maslyakova, Nikita A. Navolokin, Vadim D. Genin, Elina A. Genina, Valery V. Tuchin

**Affiliations:** 1 Core Facility Center, Saratov State Medical University, 112 Bol′shaya Kazachya Str., 410012 Saratov, Russia; gmaslyakova@yandex.ru (G.N.M.); nik-navolokin@yandex.ru (N.A.N.); 2 Science Medical Center, Saratov State University, 83 Astrakhanskaya Str., 410012 Saratov, Russia; versetty2005@yandex.ru (V.D.G.); eagenina@yandex.ru (E.A.G.); tuchinvv@mail.ru (V.V.T.); 3 Laser Molecular Imaging and Machine Learning Laboratory, Tomsk State University, 36 Lenin′s Av., 634050 Tomsk, Russia; 4 Nanobiotechnology Laboratory, Institute of Biochemistry and Physiology of Plants and Microorganisms RAS, FRC “Saratov Scientific Centre of the Russian Academy of Sciences”, 13 Prospekt Entuziastov, 410049 Saratov, Russia; khlebtsov_b@ibppm.ru; 5 Institute of Precision Mechanics and Control, FRC “Saratov Scientific Centre of the Russian Academy of Sciences”, 24 Rabochaya Str., 410028 Saratov, Russia

**Keywords:** gold nanoparticles, oncology, plasmonic photothermal therapy, photodynamic therapy

## Abstract

Cancer remains one of the leading causes of death in the world. For a number of neoplasms, the efficiency of conventional chemo- and radiation therapies is insufficient because of drug resistance and marked toxicity. Plasmonic photothermal therapy (PPT) using local hyperthermia induced by gold nanoparticles (AuNPs) has recently been extensively explored in tumor treatment. However, despite attractive promises, the current PPT status is limited by laboratory experiments, academic papers, and only a few preclinical studies. Unfortunately, most nanoformulations still share a similar fate: great laboratory promises and fair preclinical trials. This review discusses the current challenges and prospects of plasmonic nanomedicine based on PPT and photodynamic therapy (PDT). We start with consideration of the fundamental principles underlying plasmonic properties of AuNPs to tune their plasmon resonance for the desired NIR-I, NIR-2, and SWIR optical windows. The basic principles for simulation of optical cross-sections and plasmonic heating under CW and pulsed irradiation are discussed. Then, we consider the state-of-the-art methods for wet chemical synthesis of the most popular PPPT AuNPs such as silica/gold nanoshells, Au nanostars, nanorods, and nanocages. The photothermal efficiencies of these nanoparticles are compared, and their applications to current nanomedicine are shortly discussed. In a separate section, we discuss the fabrication of gold and other nanoparticles by the pulsed laser ablation in liquid method. The second part of the review is devoted to our recent experimental results on laser-activated interaction of AuNPs with tumor and healthy tissues and current achievements of other research groups in this application area. The unresolved issues of PPT are the significant accumulation of AuNPs in the organs of the mononuclear phagocyte system, causing potential toxic effects of nanoparticles, and the possibility of tumor recurrence due to the presence of survived tumor cells. The prospective ways of solving these problems are discussed, including developing combined antitumor therapy based on combined PPT and PDT. In the conclusion section, we summarize the most urgent needs of current PPT-based nanomedicine.

## 1. Plasmonic Tuning of Au Nanoparticles for Plasmonic Photothermal Therapy

### 1.1. Optical Cross-Sections and Plasmonic Tuning

When the incident light encounters a plasmonic nanoparticle, it is scattered and absorbed, thus causing the extinction of the incident light. From a quantitative point of view, all three processes can be conveniently characterized by the absorption, scattering, and extinction cross-sections $C_{abs}$, $C_{sca}$, and $C_{ext}$, which are the ratio of the corresponding powers $W_{abs,sca,ext}$ (in [W]) to the incident light intensity $I_{0}$ (in [W/cm^2^]) [1]. Therefore, the integral cross-sections have the dimension of the area and mean the corresponding energies. Due to the energy conservation, the following equality holds $\;C_{ext}\;=C_{abs}\;+C_{sca}$. The oscillating electric field displaces the metal-free electrons and creates uncompensated charges near the particle surface and the oscillating restoring forces. Owing to collective behavior, the resonance frequency of such an oscillator does not coincide with the light angular frequency ω. In fact, the collective resonance frequency is determined by many factors most important are the particle shape and structure. In contrast, the size and dielectric environment play a minor role.

Light absorption is a key process for PPT applications because of the time-efficient and nanoscale-sized transformation of electromagnetic power into heat. In short, the underlying physics can be described as follows [2]. The absorbed laser pulse quickly creates, on a time scale less than 100 fs, a highly non-thermal distribution of electron energy. Due to electron-electron scattering, a thermal electron distribution is established with a characteristic time of less than one picosecond. Because of the low heat capacity of conduction electrons, enormous electron temperatures (~1000–5000 K) can easily be reached in the nanoparticles even with a low pulse energy of ~100 nJ. The thermalized hot electrons produce lattice heating owing to electron–phonon (e-ph) processes within 1–5 ps. Typically, the particle temperature is in the order of tens of degrees higher than the initial one. The lattice heating is accompanied by its cooling due to the dissipation of heat from a particle to the surrounding medium. As the nanoparticle size is less than 100 nm, the environmental heating is strongly localized around the particle on a nanometer scale. This property is crucial for targeted and localized PPT without damage to healthy cells and tissue.

For in vivo PPT applications, the following two optical parameters of plasmonic nanoparticles should be adequately tuned: (1) plasmon resonance (PR) wavelength and (2) the scattering/absorption $C_{sca}/C_{abs}$ or the absorption/extinction $C_{abs}/C_{ext}$ ratio. To avoid unwanted absorption of light by water and hemoglobin and the scattering by inhomogeneous tissue structures, one has to use NIR or SWIR radiation. To ensure a maximal conversion efficiency of light to heat, one has to design nanoparticles with a minimal $C_{sca}/C_{abs}$ ratio. In the electrostatic approximation [1], the scattering and absorption cross-sections are

(1) Cabs=4πkIm(α), Csca=8π3k4α2,

where α is electrostatic polarizability [1] and k is the wave number in the medium. Principles of the spectral tuning can be explained by the following general expression, which relates the PR wavelength $\lambda_{PR}$ with the particle structure function $\varphi_{s}$ [3]

(2) $$\lambda_{PR}=\lambda_{p}\sqrt{\varepsilon_{ib}+\varphi_{s}\varepsilon_{m}}$$

where $\lambda_{p}=2\pi c/\omega_{p}$ is the wavelength of the volume electron plasma oscillations, $\varepsilon_{ib}$ is the interband parameter [2], $\varepsilon_{m}$ is the dielectric function of the external medium. For gold nanoparticles, $\lambda_{p}\sim 130$ nm, $\varepsilon_{ib}\sim 12$ and for the water-like environment $\varepsilon_{m}=1.33^{2}=1.77$. Therefore, the only tuning parameter is the particle structure function $\varphi_{s}$. The expression (2) was obtained [3] by analyzing the poles of analytical solutions for the electrostatic polarizability entering Equation (1). For spherical particles of a radius a, we have [3].

(3) $$\varphi_{0}(k_{PR}a)=2+\frac{12{\left(k_{PR}a\right)}^{2}}{5}\left[1+{\frac{\left(\varepsilon^{\prime \prime }/\varepsilon_{m}\right)}{24}}^{2}\right]$$

where $k_{PR}=2\pi \sqrt{\varepsilon_{m}}/\lambda_{PR}$. As the size parameter $k_{PR}a<1$, $\varphi_{s}\sim const=2$, and Equation (3) explains a weak dependence of the PR wavelength on the particle size and weak plasmonic tuning. By contrast, for spheroidal nanorods with semiaxes (b,b,a) and a>b, the particle structure function $\varphi_{s}$ reads [3]

(4) $$\varphi_{s}^{a,b}=\frac{1}{L_{a,b}}-1$$

where $L_{a,b}$ are the geometrical depolarization factors [1] that satisfy the following relations

(5) $$L_{a}=\frac{1}{h^{2}-1}\left(-1+\frac{1}{e}\mathrm{arctanh}(e)\right)$$

(6) $$L_{a}+2L_{b}=1$$

where h=a/b is the aspect ratio, $e=\sqrt{h^{2}-1}/h$ for prolate spheroids with h>1 and $e=ie_{1}=i\sqrt{h^{-2}-1}$ for oblate particles with h≤1. In the last case, arctanh(e)/e  in Equation (5) should be replaced with $\mathrm{arctg}\left(e_{1}\right)/e_{1}$. Thus, the longitudinal PR of plasmonic nanorods can easily be tuned across the Vis-NIR band by varying the aspect ratio a/b, as $L_{a}^{-1}\to \infty$ for needles and $L_{b}^{-1}\to \infty$ for thin disks. Similarly, the PR wavelength of silica/Au nanoshells moves from visible to NIR when the ratio shell/core decreases at a constant core diameter and thinner shell, or at a constant shell thickness with increasing the core diameter.

The above analysis is limited by analytical solutions for the electrostatic polarizability of small particles with a particular shape (spheres, shells, and spheroids). To avoid these limitations, Yu et al. [4] developed a universal approach based on the modal expansion of Poisson′s equation for the electric field. Evidently, this approach is still limited to small particle size as the retardation effects are neglected. However, the developed formalism can be, at least in principle, applied to the particles of arbitrary shape. In a sense, the modal expansion is similar to the so-called “ε-decomposition”, as described in [5]. Finally, in order to go beyond the electrostatic approximation, the authors of [4] developed a correction for retardation effects:

(7) $$\alpha =\frac{\varepsilon_{m}}{4\pi }\sum_{j}V_{j}B_{j}(s){\left(\frac{1}{\varepsilon /\varepsilon_{m}-1}-\frac{1}{\varepsilon_{j}-1}-A_{j}(s)\right)}^{-1}$$

where $V_{j}$ is the mode volume (the sum of $V_{j}$ equals to the particle volume V), $\varepsilon_{j}$ are the real eigenvalues corresponding to eigenmodes $E_{j}(r)$, $A_{j}(s)$ and $B_{j}(s)$ are the functions of the perturbation parameter $s=\sqrt{\varepsilon_{m}}d/\lambda$, d is the characteristic particle size. In Equation (7), the functions $A_{j}(s)$ account for the retardation effects. Inserting the retardation-corrected polarizability (7) into Equation (1) gives the absorption and scattering cross-sections of arbitrarily shaped particles. The comparison of the above solution with the exact numerical simulations for variously shaped particles demonstrated good practical accuracy for the particle size below 50–100 nm. For details, the readers are referred to Ref. [4].

Another approach is related to the modified long-wavelength approximation (MLWA) (see, e.g., [6] and references therein). For spheroids embedded in lossless or absorbing media, the improved electrostatic approximation (IEA) reads [7]

(8) $$\beta_{i}^{IEA}=\beta_{i}^{EA}\frac{1}{1-\Omega_{i}x^{2}-i\frac{2}{3h^{2}}\beta_{i}^{EA}x^{3}},\;i=a,b$$

where $\beta_{i}^{EA}=(4\pi /3V)\alpha_{i}^{EA}$ are the normalized electrostatic polarizabilities

(9) $$\beta_{i}^{EA}=\frac{\varepsilon_{r}-1}{3+3L_{i}(\varepsilon_{r}-1)},\;i=a,b$$

$\varepsilon_{r}=\varepsilon /\varepsilon_{m}$, $\varepsilon =n^{2}$ and $\varepsilon_{m}=n_{m}^{2}$ are the complex optical dielectric functions of particles and external medium, respectively; $x=ka=2\pi n_{m}a/\lambda .$ The dynamic depolarization factors $\Omega_{a,b}$ in Equation (8) are defined as follows [8]:

(10) $$\Omega_{a}=\frac{9e^{2}}{25}+\frac{\varepsilon_{r}(1-e^{2})-2}{5\left[1+(\varepsilon_{r}-1)L_{a}\right]}$$

(11) $$\Omega_{b}=-\frac{12e^{2}}{25}+\frac{\varepsilon_{r}+3e^{2}-2}{5\left[1+(\varepsilon_{r}-1)L_{b}\right]}$$

Extensive simulations for Au and Ag prolate and oblate spheroids, cigar-like rods, and disks showed excellent agreement between the absorption, scattering, and extinction cross-sections calculated by IEA and exact T-matrix method for spheroids in various dielectric and absorbing host media [7,8].

Let us now discuss the scattering albedo. It is instructive to consider first the simplest case of a small plasmonic sphere, for which we have [3]

(12) Csca/Cabs=2(ka)3β2Im(β), β=ε−εmε+2εm

To make the ratio $C_{sca}/C_{abs}$ smaller, one has to fabricate particles of small size with the optical size parameter, say ka<0.3. For example, for a typical PR laser wavelength of 520 nm and a water environment $k=2\pi \sqrt{\varepsilon_{m}}/520=0.016\;{\mathrm{nm}}^{-1}$, the sphere radius should be less than 20 nm. In the case of AuNRs, the PPT efficiency was thoroughly investigated by Lee and El-Sayed [9]. Recently, Mackey et al. investigated the PPT efficiency of AuNRs with different dimensions [10]. The authors compared three nanorod samples (38 × 11, 28 × 8, and 17 × 5 nm) and found the 28 × 8 nm AuNRs to be most effective in terms of a compromise between the absorption cross-section and the laser power converted to heat. The second important parameter is the local field extension, which ensures the effective heating after aggregation of AuNRs.

### 1.2. Generating Heat with Plasmonic Particles

To simplify consideration, we consider the laser-induced generation of heat by a single Au nanosphere of a radius a=R in water. Under the laser irradiation, the heat power density inside the sphere is given by

(13) q(r)=ω8πIm(ε)E(r)2

**Scheme 1:**
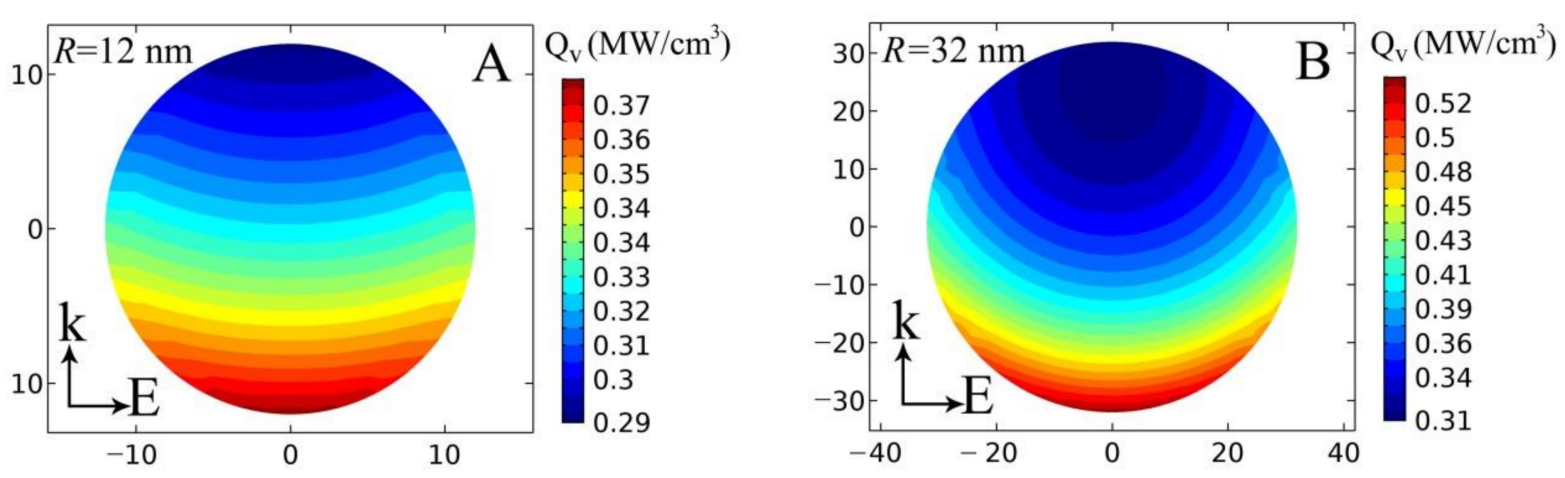
Two-dimension mapping of the power-density $Q_{V}=q$ distribution in Au spheres under plasmon resonance excitation in the air (~510 nm). The sphere radii are 12 nm (**A**) and 32 nm (**B**), the incident intensity is 1 W/cm^2^. The calculations were made by S. Zarkov (IBPPM RAS and IPMC RAS).

The inner field and the heat power density are homogeneous in the electrostatic limit. However, with an increase in the particle size, the retardation effects lead to nonhomogeneous field distribution, especially at CW irradiation. If we consider a cross-section perpendicular to the wave vector, the retardation effects disappear. However, in a plane containing the wave vector, the retardation effects and inhomogeneous power density distribution are clearly seen even for small sphere radii 12 and 32 nm (Scheme 1).

Fortunately, because of the high ratio of thermal conductivity of gold and water (~530), the temperature distribution inside the particle is highly homogeneous even though the heat source distribution is not [11]. Thus, one can only consider the power density as a function of time. In the case of CW illumination, the total heat Q (in [W]) dissipated in the particle is $Q=\int qdV=C_{abs}I_{0}$, where $I_{0}$ is the incident light intensity (usually, in [W/cm^2^]).

The heat exchange between the particle and the external medium (here, water) is described by the heat diffusion equation

(14) $$c_{i}\rho_{i}\frac{\partial T}{\partial t}=\kappa_{i}\nabla^{2}T+q_{i},\;i=g,w$$

where the indexes i=g and i=w stand for gold and water, respectively, *C*_*i*_ is the specific capacity at constant pressure, $\rho_{i}$ is the mass density, $\kappa_{i}$ is the thermal conductivity, $q_{g}=q$ for r≤R, and $q_{w}=0$ for r>R outside the particle. The heat diffusion equation should be supplemented by the boundary conditions for temperature and its radial derivatives [12,13].

It follows from the dimension analysis of Equation (14) [12,13] that two characteristic times define the temperature evolution inside the particle, $\tau_{g}\sim R^{2}/D_{g}$ and in water $\tau_{w}\sim R^{2}/D_{w}$, where D=κ/ρc is the thermal diffusivity. For gold and water $D_{g}=1.27$ and $D_{w}=0.00143$${\mathrm{cm}}^{2}/s$, respectively. Therefore, $\tau_{w}\gg \tau_{g}$ and the overall temperature evolution is governed by $\tau_{w}$~ 0.1 ns and 1 ns for the particle diameters 10 and 100 nm, respectively [14]. Thus, the steady-state temperature distribution establishes very fast in the case of nanoparticle heating. To evaluate the final temperature profile, one can consider the Poisson Equation (14) with the right part equal to zero. In the case of CW illumination, the temperature increase in water is

(15) $$\Delta T^{CW}(r)=\Delta T_{p}^{CW}\frac{R}{r},\;r\geq R$$

where the temperature increase in the particle (or, roughly equivalently, on the particle surface) is calculated by [13]

(16) $$\Delta T_{p}^{CW}=\frac{C_{abs}I_{0}}{4\pi \kappa_{w}R}\left[1+\frac{\kappa_{w}}{2\kappa_{g}}\left(1-\frac{r^{2}}{R^{2}}\right)\right]≃\frac{C_{abs}I_{0}}{4\pi \kappa_{w}R},\;r\leq R$$

where the last expression was obtained due to condition $\kappa_{w}/\kappa_{g}\sim 0.002$. Note that the temperature increase in a small particle scales as $R^{2}$ because $C_{abs}\sim V\sim R^{3}$. Furthermore, Equation (16) can be roughly applicable to various particle shapes provided that an appropriate correction shape factor 1<β<2 is introduced into the denominator [15]. From Equation (4), the maximal temperature increase is negligible (<0.1 K) for all particles with R<50 nm under CW irradiation with typical intensities $I_{0}\sim 1-10$ W/cm^2^ [16]. A typical absorption cross-section of gold nanorods is of the order 2000 nm^2^. At irradiance 2 W/cm^2^, the absorbed power is 4 × 10^−11^ W and the maximal temperature increase from Equation (16) is ~0.001 K. Therefore, CW irradiation cannot produce any significant local heating. However, if we consider an ensemble of particles in a volume $V_{L}\sim L^{3}$, the global temperature increase [17] can be evaluated as $\Delta T_{global}/\Delta T_{p}={\left(L/R\right)}^{2}f$ with the filing factor $f=(4\pi R^{3}/3)N$. This expression can be recast as $\Delta T_{global}/\Delta T_{p}=VN(R/L)=N_{tot}(R/L)$. For a typical NP concentration $N\sim 10^{11}$ cm^−3^ and $R/L=10\;\mathrm{nm}/1\;\mathrm{cm}=10^{-6}$, the above ratio equals 10^5^ and the global temperature increase is ~100 K. This estimation agrees with theoretical [18] and experimental data (see below).

If the particle/water interface has a nonzero Kapitza resistivity 1/G [13], the continuity condition for temperature is replaced by

(17) $$-\partial_{r}T(R^{+},t)=l_{K}^{-1}\Delta T(t)=l_{K}^{-1}[T(R^{+},t)-T(R^{-},t)]$$

with the Kapitza length *l_K_* = *K*_w_/*G*. The interface resistivity can be neglected if the Kapitza number g*_K_* = *l_K_*/R is small. For Au spheres with diameters from 10 to 100 nm, the Kapitza number $g_{K}$ varies from 2 to 0 for the water environment. Another important example is a gold nanorod with CTAB coating. For typical 50 × 12 nm dimensions, $g_{K}$ is about 0.4 [14]. Finally, an additional coating of nanoparticles with an outer shell introduces quite a different interface and will greatly affect the thermoplasmonic properties [19]. For nonzero $g_{K}$, Equation (16) can be generalized by replacing the first term 1 in the squared brackets with $1+g_{K}$ [13].

Consider now the steady-state regime at pulsed laser irradiation. This regime involves three stages: (1) the electronic absorption and thermalization (~100 fs); (2) electron-phonon (e-ph) thermalization, which does not depend on the particle size and takes about 1.7 ps; (3) External heat diffusion, which characteristic time ranges from 0.1 ns to a few ns depending on the gold particle size and interface properties. These estimations agree with reported experimental data for metal nanoparticles in water and organic solvents [20,21]. Because of fast e-ph thermalization as compared to stage 3, the maximal temperature increase for pulsed irradiation can be estimated as follows [13]

(18) $$T_{p}^{0}=\frac{C_{abs}F}{V\rho_{g}c_{g}}=\frac{C_{abs}<I_{0}>}{V\rho_{g}c_{g}f_{p}}$$

where $F=<I_{0}>/f_{p}$ is fluence at a given average irradiance $<I_{0}>$ and repetition rate $f_{p}$. For an Au particle (*R* = 10 nm), the absorption cross-section in water is 400 nm^2^. Then, at a fluence 0.1 mJ/cm^2^ the temperature increase is ~40 K. This example demonstrates the significant advantage of pulsed heating compared to CW irradiation. Therefore, for small particles $C_{abs}\sim V$, the temperature increase does not depend on the particle size, in contrast to CW case where the temperature increase scales as $R^{2}$. Note also that simple Equation (18) is applicable to nonspherical particles also.

The time-dependent evolution of the particle temperature is described by a stretched exponential function (1/G = 0 is assumed) [13]

(19) $$T(R,t)\sim \exp\left[-{\left(\frac{D_{w}t}{0.041R^{2}}\right)}^{0.39}\right]$$

Numerical simulations of the steady-state temperature profiles for CW and pulsed irradiation showed much stronger temperature localization in the last case. In particular, for pulsed irradiation, the envelope of the spatial temperature profile is given by [13]

(20) $$T_{p}^{pulsed}(r)\sim \exp\left[-{\left(\frac{r-R}{0.06R}\right)}^{0.45}\right]$$

whereas for CW illumination $T_{p}^{CW}(r)\sim r/R$. The above dependencies were obtained under two assumptions: (1) the initial temperature increase is instantaneous and homogeneous; (2) the temperature profile inside the particle remains homogeneous during all stages. It has been shown that both approximations are in reasonable agreement with exact numerical simulations [13]. Introducing a finite interface conductance 1/G results in significant modifications of results obtained for the ideal model (see Figure 7 in [13]). The main conclusions are as follows: (1) There exist an optimal particle diameter 40 nm, when the particle is an effective absorber, and the temperature release is not so fast. For a particular case ($f_{p}=$ 86 MHz, $<I_{0}>=$ 10^3^ mW/cm^2^), the maximal temperature increase of a 40 nm particle ranges from 40 to 46 K for all nanoparticle surface conductivities G from 50 (MW/m^2^/K) to infinity. By contrast, the maximal water temperature increase strongly depends on G and R, and for 100 nm particles, it is close to 0. (2) The interface thermal resistivity increases the particle temperature and decreases the heat release in the water. (3) The pulsed illumination results in strong temperature confinement at nanoscale compared to CW illumination. (4) For large nanoparticles (diameter > 50–100 nm), the pulse illumination does not provide more efficient heating compared to the CW case, especially for high interface thermal resistance.

It should be stressed that the heating/cooling kinetics can be strongly affected by the Biot number Bi=hR/κ [22], where h=G is the thermal conductance per unit area of the interface. Specifically, Fedorenko et al. [22] reported two characteristic times $\tau_{h}=c_{V}R/3h\approx 16-32$ ms and $\tau_{D}=R^{2}/\pi^{2}D\approx 10-40$ ns for laser heating/cooling of DyPO_4_ nanocrystals in the air [23]. Therefore, for DyPO_4_ nanoparticles in air Bi≪1 and the characteristic time scale is determined by heat transfer at the boundary rather than thermal diffusivity

(21) $$\Phi (t,R)=\Phi_{s}\left[1-\exp(-t/\tau_{h}\right],\Phi_{s}=(4\pi /3)R^{3}q$$

(22) $$\Delta T(t,R)=T_{s}\;\left[\;,\;1-\exp(-t/\tau_{h}\right],T_{s}=qR/3h$$

The cooling kinetics is determined by the same exponents. Owing to different size dependence of $\tau_{h}\sim R$ and $\tau_{D}\sim R^{2}$, the size polydispersity modifies the monodisperse approximation to a different extent. Experimental data of [22] reveal even more relaxation times between 0.1–0.2 s. Such slow kinetics results from specific experimental conditions. The heat transfer conductivity $h=G=5\times 10^{-4}{(\mathrm{Wcm}}^{-2}K^{-1})$ was seven orders less than a typical minimal value $G=(5-10)\times 10^{3}{(\mathrm{Wcm}}^{-2}K^{-1})$ for gold/water interfaces [13,19,20,24].

## 2. The Main Types of Plasmonic Particles in PPT

**Figure 1:**
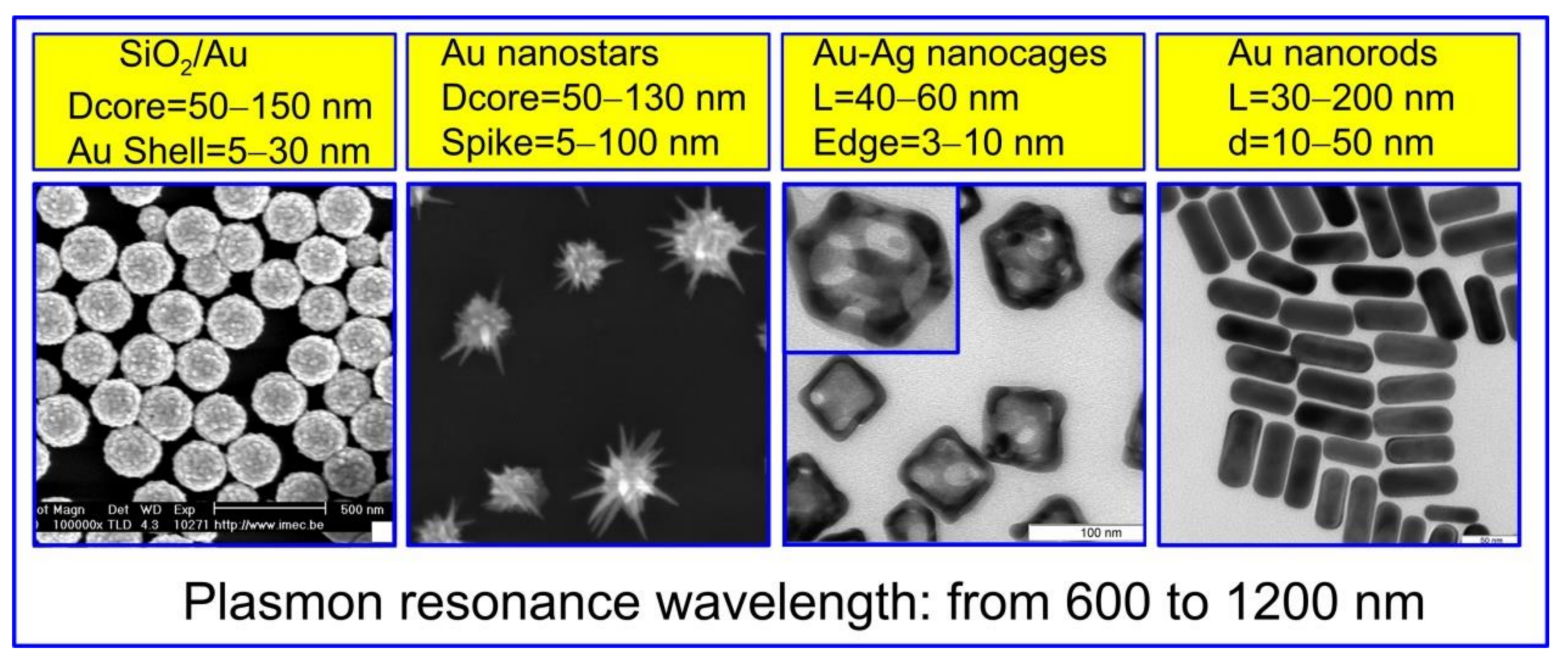
Typical TEM images and dimensions of silica(core)/Au nanoshells (AuNSHs), Au nanostars (AuNSTs), Au-Ag nanocages (AuNCGs), and Au nanorods (AuNRs). The PR wavelength varies across the Vis-NIR region from 600 to 1200 nm through the variation of the structure and shape of particles. The images were provided by Lab of Nanobiotechnology, IBPPM RAS.

At present, there is a great variety of Au or composite nanoparticles available through wet chemical synthesis [25]. However, for PPT applications, only four particle types—SiO_2_/Au nanoshells, Au nanostars, Au-Ag nanocages (AuNCGs), and Au nanorods (AuNRs) [26,27,28,29,30,31]—have found a general use for the model in vitro and in vivo experiments, owing to convenient tuning across Vis-NIR region from 600 to 1200 nm (Figure 1).

In addition to conventional NIR optical window (700–1000 nm), the second NIR-II widow (1000–1400 nm) has emerged for applications in biomedicine as a possible alternative to NIR-I window [32,33]. The rationale for such replacement is more negligible scattering of light in in vivo environment, thus requiring lower irradiation light intensity. Accordingly, the reduced power is less than the ANSI skin tolerance threshold, and the NIR-II window seems more suitable for in vivo experiments [28,33]. What is more, the maximum permissible exposure (MPE) of skin for NIR-II (1 W/cm^2^) is three times higher than that for NIR-I (0.33 W/cm^2^) [34]. That is why the NIR-II nanoparticles attracted much attention (for proper citations, the readers are referred to the review by Kim et al. [28]). Recently, we have shown both experimentally and via electromagnetic simulations [35] the existence of two plasmonic maxima (NIR-I-II and SWIR) of AuNSTs, as fabricated by the surfactant-free Vo-Dinh method [36].

### 2.1. Silica/Gold Nanoshells (AuNSHs)

**Figure 2:**
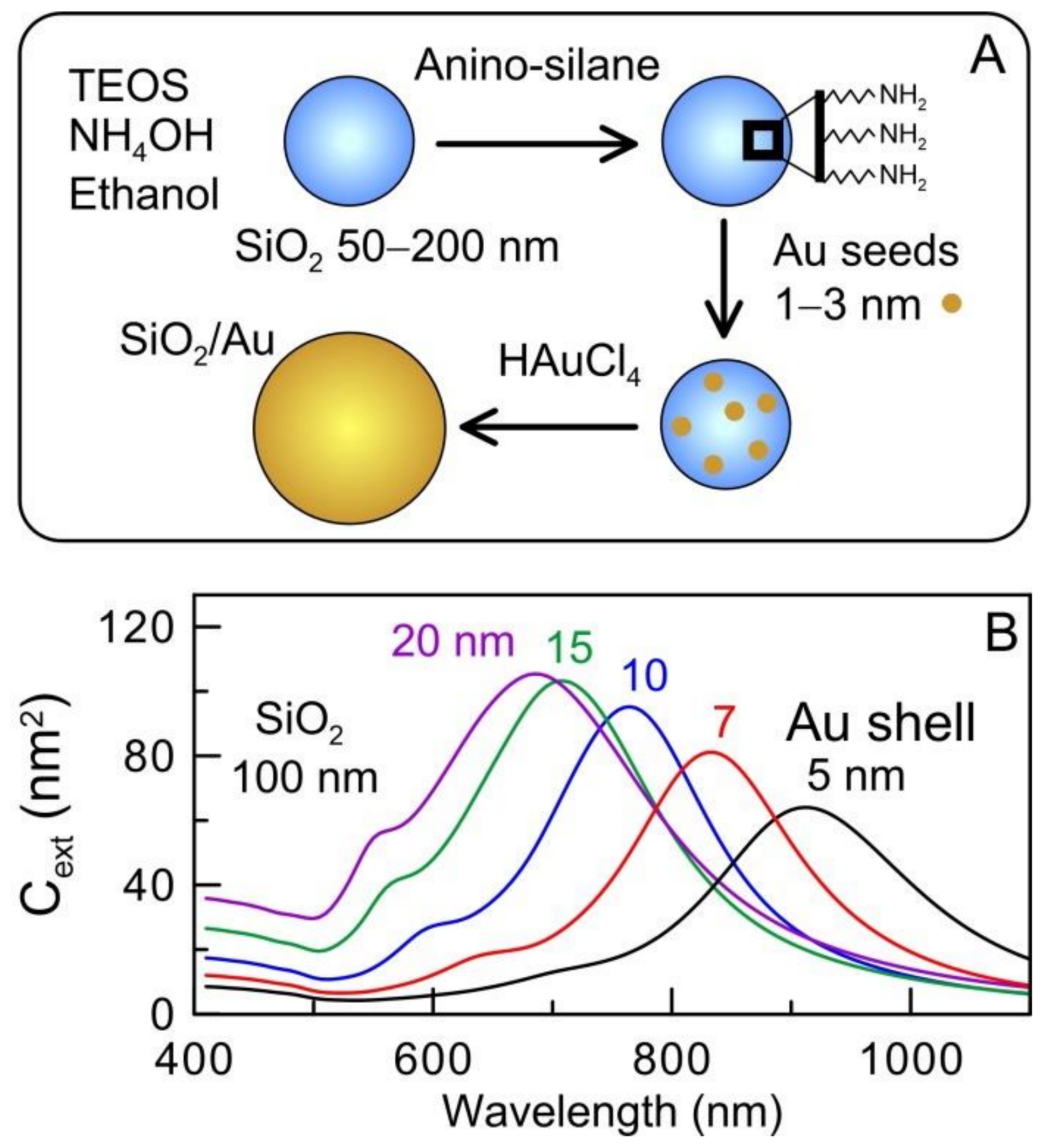
(**A**) Scheme for the synthesis of AuNSHs with a silica core. (**B**) The extinction cross-sections of AuNSHs as predicted by Mie theory for silica core 100 nm. The Au shell thickness has the normal distribution with the average values 5, 7, 10, 15, and 20 nm and the normalized standard deviation of 0.1. The size correction for Au dielectric function in the shell was included in the calculations.

Figure 2 illustrates the scheme of AuNSH synthesis, which starts from the fabrication of silica nanoparticles by hydrolysis and subsequent condensation of tetraethylorthosilicate (TEOS) in ethanol in the presence of ammonia. This process is known as the Stöber method [37]. The average diameter of silica particles is determined by the molar ratio of TEOS to ammonia and can be varied from 50 nm to 1 μm [38].

The second step in AuNSH fabrication is the surface functionalization of silica cores with positively charged groups of 3-aminopropyltrimethoxysilane (APTMS) [39]. Then, small Au seeds of 1–3 nm are adsorbed on the silica surface to form the centers for Au condensation. At the final step, the gold layer (5–50 nm) is formed owing to the reduction in HAuCl_4_ by hydrochloride or formaldehyde on adsorbed gold seeds. With the typical core diameters of 100–150 nm and shell thickness of 15–30 nm, the PR wavelength varies from 600 to 900 nm (Figure 2B). The main drawback of the resulting nanoshells is a small quality factor of spectra and the limiting value of the upper PR wavelength (typically, less than 900 nm).

The scheme in Figure 2 involves four steps. Guan et al. [40] introduced a one-step protocol consisting of the simultaneous growth of silica cores and AuNSHs using the hydrolysis of APTMS. This method produces the AuNSHs with significant polydispersity and a narrow range of plasmonic tuning between 600 and 850 nm.

Despite 15-year investigations of PTT in many laboratories [41,42], all attempts in clinical implementation have been unsuccessful. Surprisingly enough, not the best particles from the plasmonic Zoo–SiO_2_/Au nanoshells (Nanospectra Biosciences, Inc., Houston, TX, USA) are the only example for which three clinical trials have been conducted. In particular, the so-called AuroLase^®^ therapy was tested against refractory/recurrent head and neck tumors and metastatic lung tumors with blurred results [43]. A third clinical trial was started for localized prostate cancer therapy combining AuroShell light-activated tumor ablation with MR/US imaging [43]. Preliminary data show encouraging results, but the long-term efficacy and possible side effects remain to be studied [44].

### 2.2. Gold Nanostars (AuNSTs)

AuNSTs can be fabricated by seed-mediated method [45,46] and by seedless one-pot protocols [47,48]. As a rule, the seed-mediated synthesis produces more monodisperse and reproducible particles than seedless protocols [49]. In 2012, the Vo-Dinh group [50] suggested a surfactant-free synthesis of high-quality AuNSTs by using ascorbic acid Ag ions as a reductant and branch-directing agent, respectively. This approach has become most popular because of several reported improvements [51,52]. Note that all previous reports used 15 nm seed particles, thus limiting the size tuning of the final NSTs.

In a typical seed-mediated protocol, 1–3 nm seeds are prepared by the Duff method [53], as described in detail in Ref. [51]. For the synthesis of larger 15–35 nm seeds, the citrate reduction by Frens is an optimal choice [54]. For AuNST synthesis, 300 μL of 13–35 nm citrate-stabilized seeds was added to 10 mL of 0.75 mM auric chloride (HAuCl_4_) with 30 μL of 1 M HCl in a 20 mL glass vial at room temperature under moderate stirring (700 rpm). At the same time, 300 μL of 2 mM silver nitrate and 150 μL of ascorbic acid (AA; 100 mM) were quickly added. The solution was stirred for 30 s as its color rapidly turned from light red to blue or green. In the case of the smallest 3 nm seeds, the protocol is the same except for smaller 10 μL of a 3 nm seed solution added to the growing solution.

**Figure 3:**
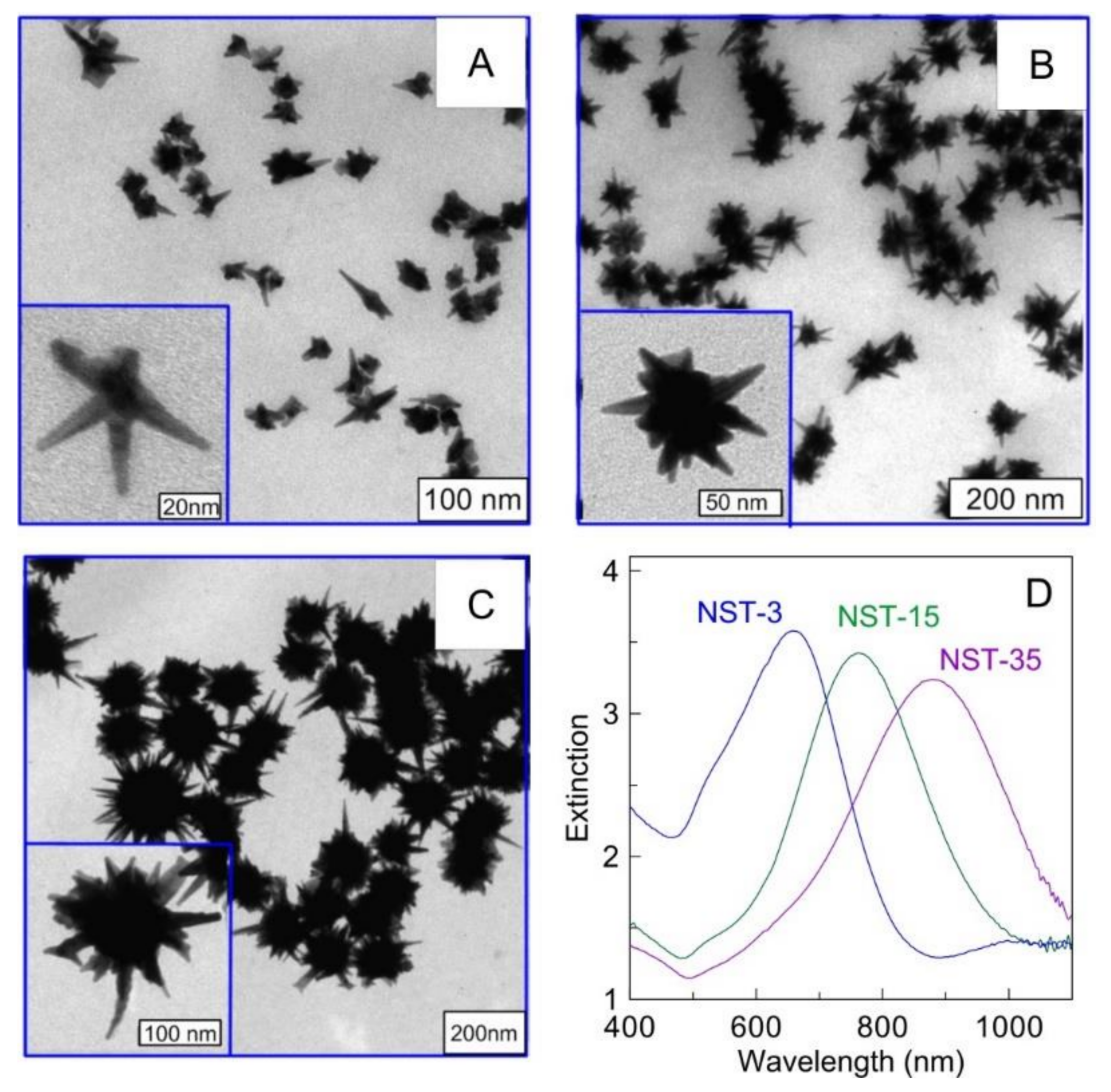
TEM images of AuNSTs fabricated with 3 nm (**A**), 15 nm (**B**), and 35 nm (**B**) seeds and the corresponding extinction spectra (**D**). The scale bars are 100 (**A**) and 200 nm (**B**,**C**). The insets show enlarged images with scale bars 20 (**A**), 50 (**B**), and 100 nm (**C**).

Figure 3 shows TEM images and extinction spectra of three AuNST samples prepared with 3, 15, and 35 nm seeds. These data demonstrate effective plasmonic tuning of AuNSTs through the variation in the size and concentration of seeds. More detailed investigations of all controlling parameters have been reported by the Vo-Dinh group [52].

Plasmonic AuNSTs have been reported as useful photothermal agents [55,56] for PTT therapy of inflammatory breast cancer tumor emboli [57], heating efficiency in PTT treatment of PC3 tumors in mice [58] (direct injection of AuNSTs was used); in the combination of PTT and immunotherapy [59,60].

### 2.3. Au-Ag Nanocages (AuNCGs)

**Figure 4:**
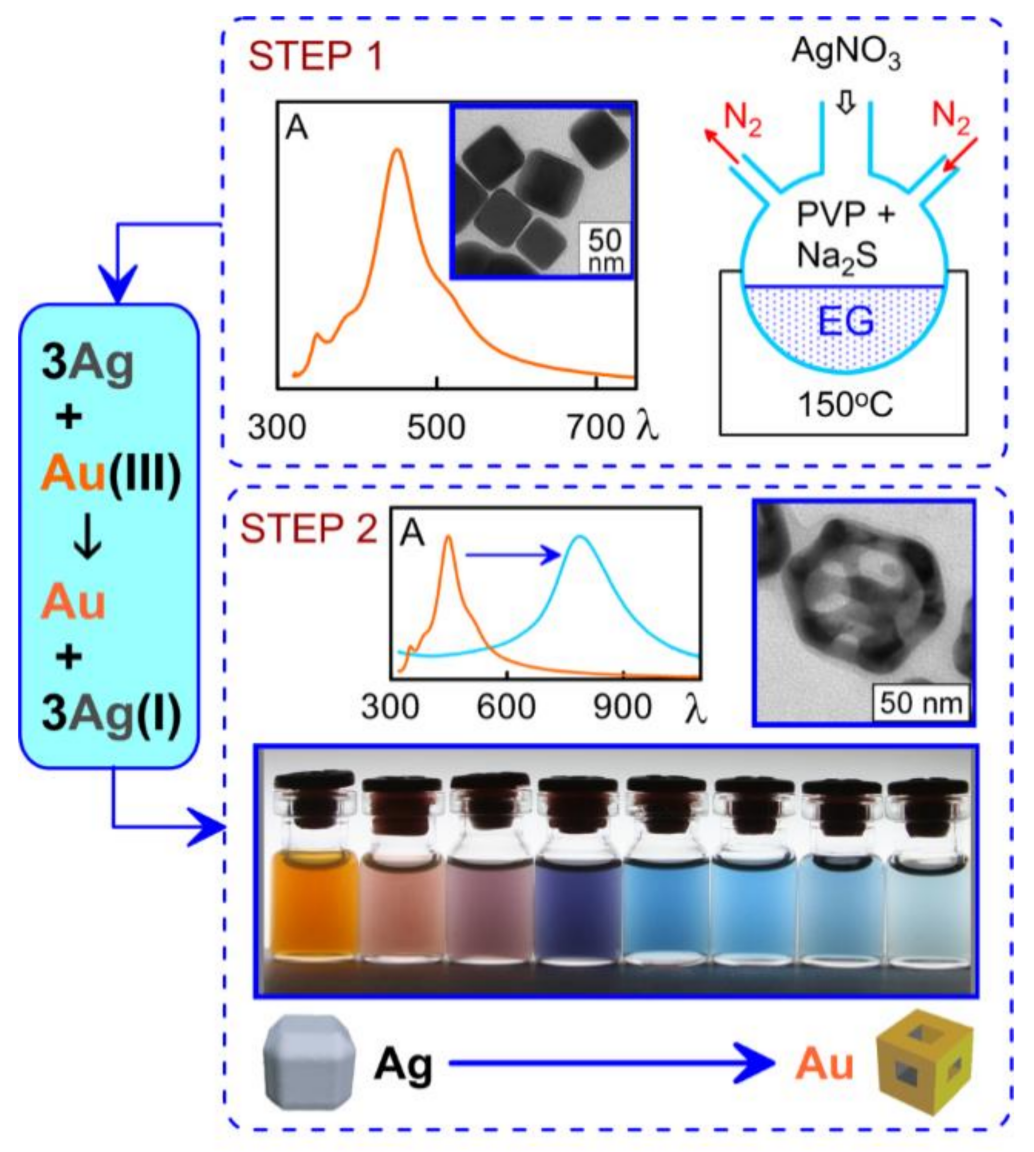
Two-step scheme for the synthesis of AuNCGs. The first step results in the formation of approximately 50 nm silver nanocubes with a characteristic plasmon resonance of about 450 nm. The second step gives the appearance of porous nanoparticles owing to the galvanic replacement process. The examples of TEM images of Ag nanocubes, changes in the colloid color, and extinction spectra in the process of synthesis are illustrated by the insets.

The synthesis of AuNCGs is a two-step process illustrated by Figure 4 [61]. The most challenging step is the synthesis of Ag nanocubes by a high-temperature polyol process as introduced by the Xia group [62,63].

**Figure 5:**
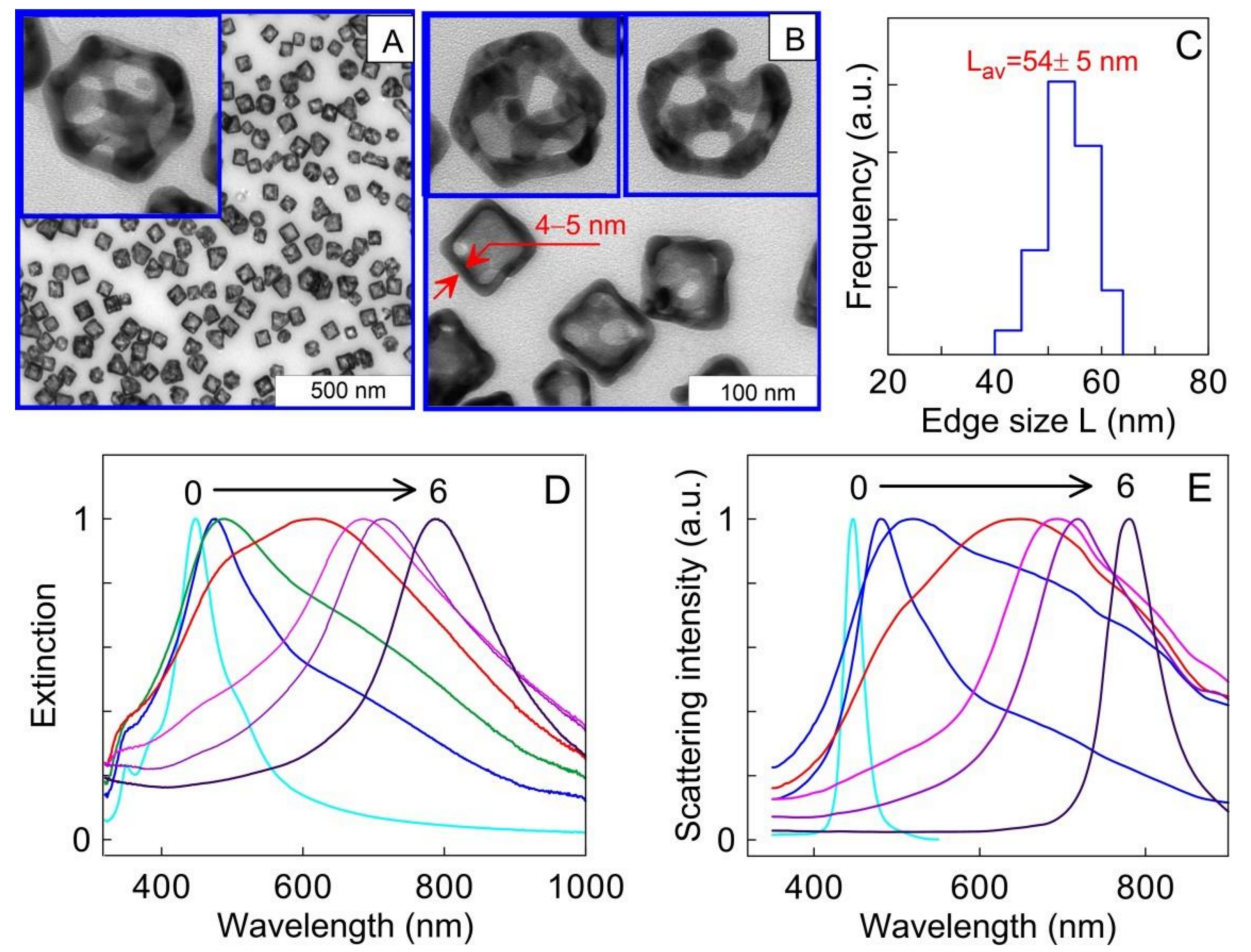
Overview (**A**) and enlarged ((**B**) and inset in (**A**)) TEM images of AuNCGs and the particle-size distribution (**C**). Various hollow and porous particles with a typical wall thickness of 4–5 nm are seen in the panel (**B**). During the galvanic replacement process, the plasmonic peaks progressively move from 420 nm to 800 nm (extinction and differential light scattering spectra in panels (**D**) and (**E**), respectively).

In the second step, the silver-gold galvanic replacement reaction is carried out by the addition of AuHCl_4_ and heating the reaction mixture for boiling temperature (100 °C). During the second step, three silver atoms are replaced with one gold atom. Therefore, this process results in progressive loss of silver mass and the formation of AuNCGs. Because of the high excess of gold ions, the hollow and porous alloyed nanostructures are formed with the continuous replacement of silver by gold. Figure 5A,B show typical TEM images of such particles called “nanocages” [64]. The typical average edge sizes of AuNCGs lie between 40 and 60 nm (Figure 5C). Depending on the particle morphology, the formation of AuNCGs is accompanied by red-shifting the extinction (Figure 5D) and elastic light scattering (Figure 5E). Note that the scattering spectra in Figure 5E were measured by the differential light scattering spectroscopy method [65].

In this case, the initial plasmon resonance of silver nanocubes in water occurs at 420 nm and then moves to 800–1000 nm, depending on the morphology of the particles (Figure 5C,D. AuNCGs have much more complex random structure than simple nanoshells on silica cores. In particular, such nanoparticles often have a cubical shape, whereas the shell and edges may contain silver atoms, pores, and cavities. Nevertheless, the galvanic replacement method gives reproducible plasmonic spectra with a characteristic peak located near 800 nm. A significant drawback of the Xia method is the complexity of the polyol synthesis of silver cube templates. Our practical expertise has shown that one polyol process must be thoroughly optimized to give reproducible synthesis [66]. Further, the galvanic displacement reaction at 100 °C requires careful control for the rate of addition of reagents. To avoid some of the noted problems, we recently proposed [67] a new technique for obtaining hollow gold particles by the synthesis of silver templates and the galvanic replacement reaction in the CTAB solution. The main advantage of this procedure is the possibility to fabricate AuNSHs and AuNCGs through the simultaneous addition of all reagents at room temperature.

After pioneering synthesis studies, the Xia group reported the first biomedical applications of AuNCG-based bioconjugates [68,69]. Last year, there appeared many reports on the application of AuNCGs to PPT therapy of cancer cells in vitro [70,71] and cancer tumors in vivo [72,73,74,75,76].

### 2.4. Au Nanorods (AuNRs)

In spite of the enormous number of species in the Plasmonic Nanoparticle Zoo, the AuNRs are the most demanded and versatile plasmonic nanoplatforms. We highly recommend to readers an excellent recent review published by J. Wang and coworkers [77]. This document contains 112 pages and 1304 references. It covers all AuNR-related studies, including synthesis, AuNR-based-heterostructures, assemblies of AuNRs, classical and quantum-related plasmonics, applications in sensing, SERS, optoelectronics, photocatalysis, nanobiotechnology, and therapeutics.

The main synthesis route for obtaining AuNRs is the seed-mediated approach [78] in which a small 1–3 nm ice-cooled seed solution is added to a growing medium containing HAuCl_4_, CTAB, and reducing agent, for example, ascorbic acid or hydroquinone. Although the first studies of wet-chemical AuNR synthesis were reported by the Murphy group [78], a real breakthrough in the synthesis technology was made in 2003 by Nikoobakht and El-Sayed [79]. They proposed two crucial improvements: (1) the use of CTAB-stabilized gold nanoclusters as seeds and (2) adding silver nitrate to the growth solution. This protocol produces high-quality AuNRs with typical thickness 10–12 nm, length 30–70 nm, and yield up to 90%.

**Figure 6:**
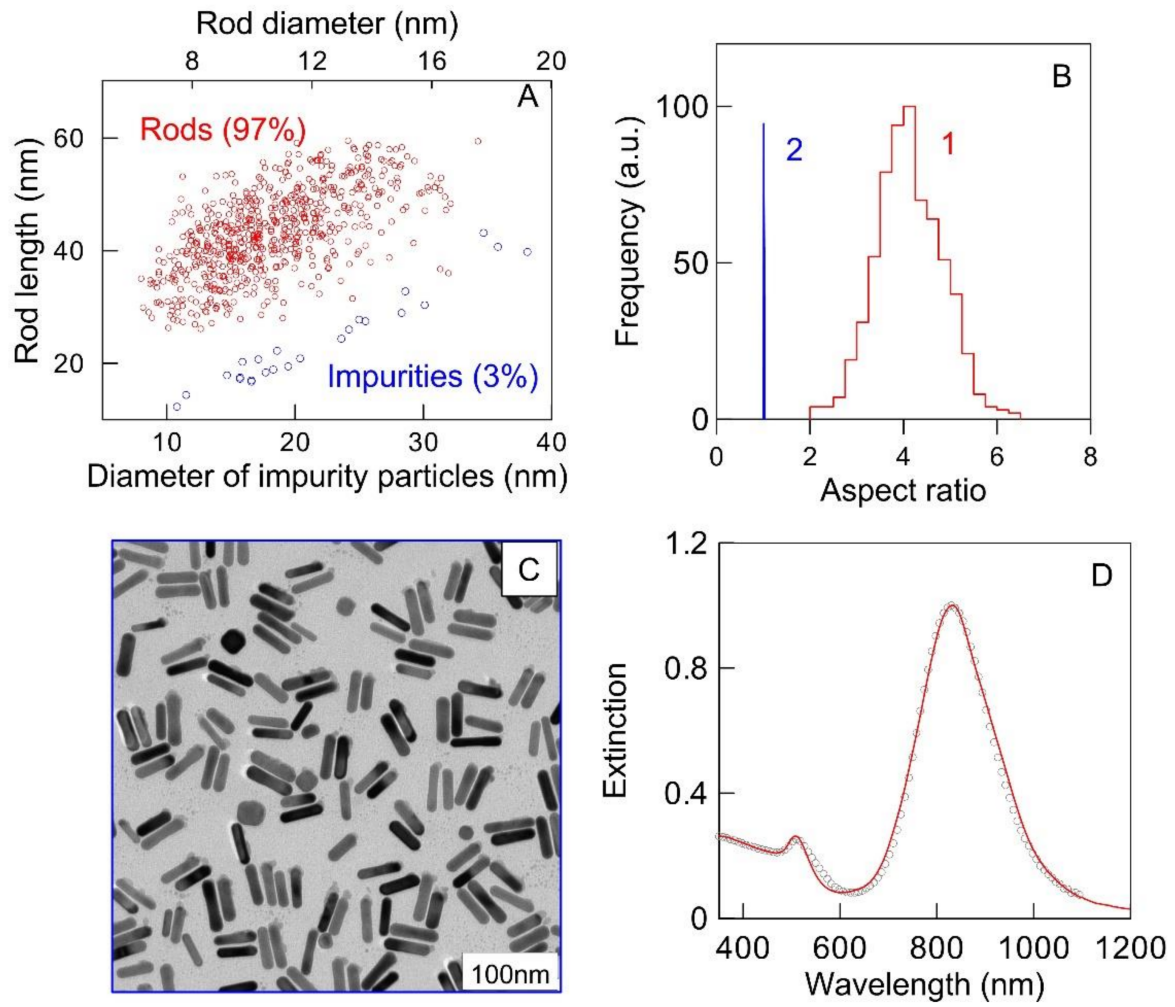
(**A**) Length-diameter distributions for the NR-830 sample, containing a major fraction of rods (97% from TEM images) and 3% of impurity particles. (**B**) The aspect ratio distributions for the rods (1) and impurities (2). (**C**) TEM image, the scale bar is 100 nm. (**D**) Experimental (circles) and T-matrix simulated spectra for the polydisperse ensemble of AuNRs (for details, the readers are referred to Ref. [80]).

Figure 6A shows 2D distributions length-diameter for AuNRs (97% by number, red points) and the impurity particles (3%, blue circles). The aspect ratio histogram (Figure 6B) shows the average aspect ratio of about 1.2 for impurities and 4 for AuNRs. Figure 6C illustrates a typical overview TEM image of AuNRs fabricated by seed-mediated protocol [79]. Finally, shown in panel D is the measured (red line) and simulated (points) extinction spectra. Details of the T-matrix simulations can be found in Ref. [80].

Currently, the seed-mediated CTAB-assisted protocol [79] is a “gold standard” for the synthesis of AuNRs in all laboratories. However, it is not free from such drawbacks as the limited spectral and geometrical tuning and, in some cases, a notable percentage (~10%) of impurities. In 2013, Murray and coworkers [81] suggested a new fabrication method based on the use of a binary surfactant mixture (CTAB and sodium oleate, NaOL).

In our work [82], the Murray method was slightly modified to use the AuNRs for further overgrowing for desired geometrical parameters. Specifically, it is possible to synthesize particles with a thickness ranging from 12 to 100 nm, the axial ratio from 2 to 10, and the PR wavelength from 600 to 1300 nm. Vigderman and Zubarev [83] suggested using hydroquinone to reduce Au ions. With this protocol, it is also possible to obtain long AuNRs with PR of more than 1200 nm. Finally, several groups reported “seedless” methods, in which the AuNR synthesis is carried out in one-pot step [84,85,86,87]. It should be noted here that the “seedless” term is not entirely correct because, after the addition of NaBH_4_ to the reaction mixture, small gold nanoclusters are instantly formed to serve as condensation centers during the further reaction.

**Figure 7:**
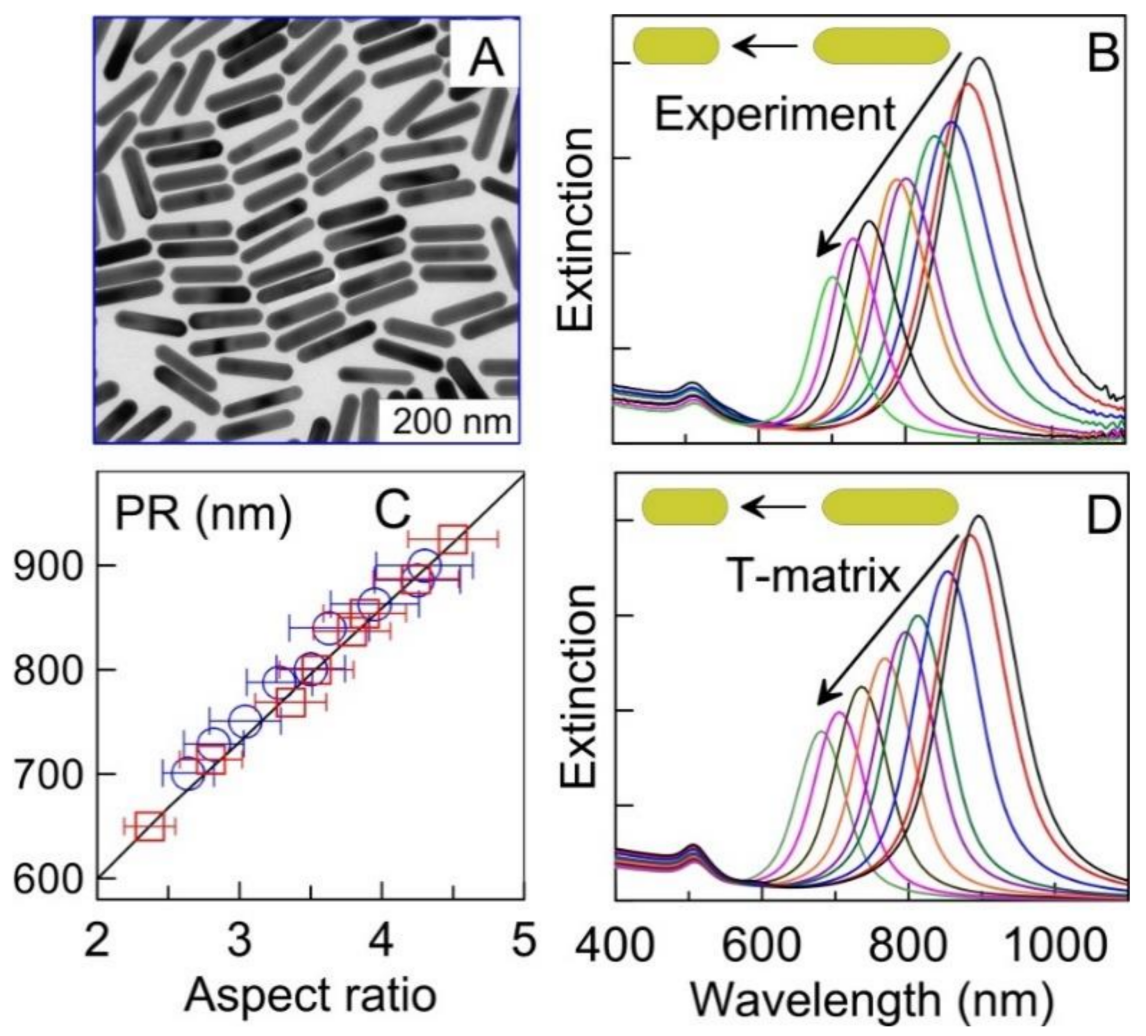
TEM image of AuNRs with PR wavelength of 920 nm. (**A**). Panels (**B**,**D**) show experimental and simulated extinction spectra of 9 samples during the etching process. Panel (**C**) displays a linear dependence between PR wavelength and TEM-derived average aspect ratio. Data for independent runs are sown together with STD errors. The line shows the T-matrix dependence calculated for polydisperse TEM ensembles (for details, see Ref. [89]).

Both seed-mediated and seedless protocols have limited accuracy for PR tuning from run to run synthesis. As a rule, the PR wavelength can be reproduced with 20 nm accuracy. Recently, we suggested two practical protocols for fine-tuning the localized PR wavelength and the particle volume, which determines the absorption/scattering ratio. The protocols are based on a combination of overgrowth [82] and etching [88] processes. The overgrowth and etching technologies can be used for the rational design of rod geometry to control the localized PR wavelength and the optical absorption/scattering ratio. Figure 7 shows the TEM image of the initial rods with PR wavelength 920 nm. During the nanorod etching, the rod length can be precisely reduced while the rod diameter remains constant. As a result, the progressive decrease in the aspect ratio and PR wavelength is observed (Figure 7B–D). Note the remarkable agreement between measured and simulated extinction spectra and excellent reproducibility of the linear correlation between the measured PR wavelengths and the aspect ratio of rods. Furthermore, the T-matrix prediction (line in panel C) is in excellent agreement with measured data.

To date, the AuNRs are the most popular nanoparticles in all biomedical applications, including PPT with various nanorod-based synergistic platforms [90,91]. In particular, AuNRs were conjugated with DARPin_9–29 [92], peptides [93], polyTLR7/8a [94], cell membrane [95], albumin [96], and other substances. AuNRs were incorporated into mesoporous silica [97,98], biodegradable polymeric matrix [99], and liposomes to facilitate the targeted release of ruthenium(II) polypyridyl complexes [100]. For more detailed information, the readers are referred to resent original papers [101,102,103,104,105,106,107,108,109] and related citations in recent reviews [77,110,111,112,113,114,115].

### 2.5. Comparison of PTT Efficiency of Different Plasmonic Nanoparticles

To enhance the PPT efficiency, it is crucial to make the right choice of nanoparticles with maximal photothermal conversion at minimal particle dose. Perhaps, the Halas group was the first who address the problem by using silica/gold and Au_2_S/Au nanoshells and AuNRs as examples [116]. Roper et al. [117] developed a simple theoretical model for quantitative evaluation the PTT efficiency *η* by using PTT experiments with suspensions

(23) $$\eta =\frac{AS(T_{\max}-T_{amb})-Q_{0}}{I_{0}(1-10^{-A_{\lambda }})}$$

where A is the proportionality constant (to be determined separately), S is the area cross-section perpendicular to conduction, $T_{\max}$ is the maximum equilibrium temperature, $T_{anb}$ is the ambient temperature of the surrounding, $Q_{0}$ represents heat dissipated from light absorbed by the measuring system itself, $I_{0}$ is the incident intensity, and $A_{\lambda }$ is the absorbance at a given wavelength. It is this simple model that was used by the Halas group for comparative measurements. As the concentration of particles varied within two orders, the authors used equivalent optical density at 815 nm for all three suspensions. The experimental photothermal efficiency as calculated by Equation (23) was highest for AuNRs and minimal for Si_2_O/Au nanoshells. However, the photothermal efficiency of a single particle η was highest for Si_2_O/Au nanoshells. Note that the parameter η was calculated by multiplying the empirical efficiency of a single particle by the calculated extinction cross-section.

**Figure 8:**
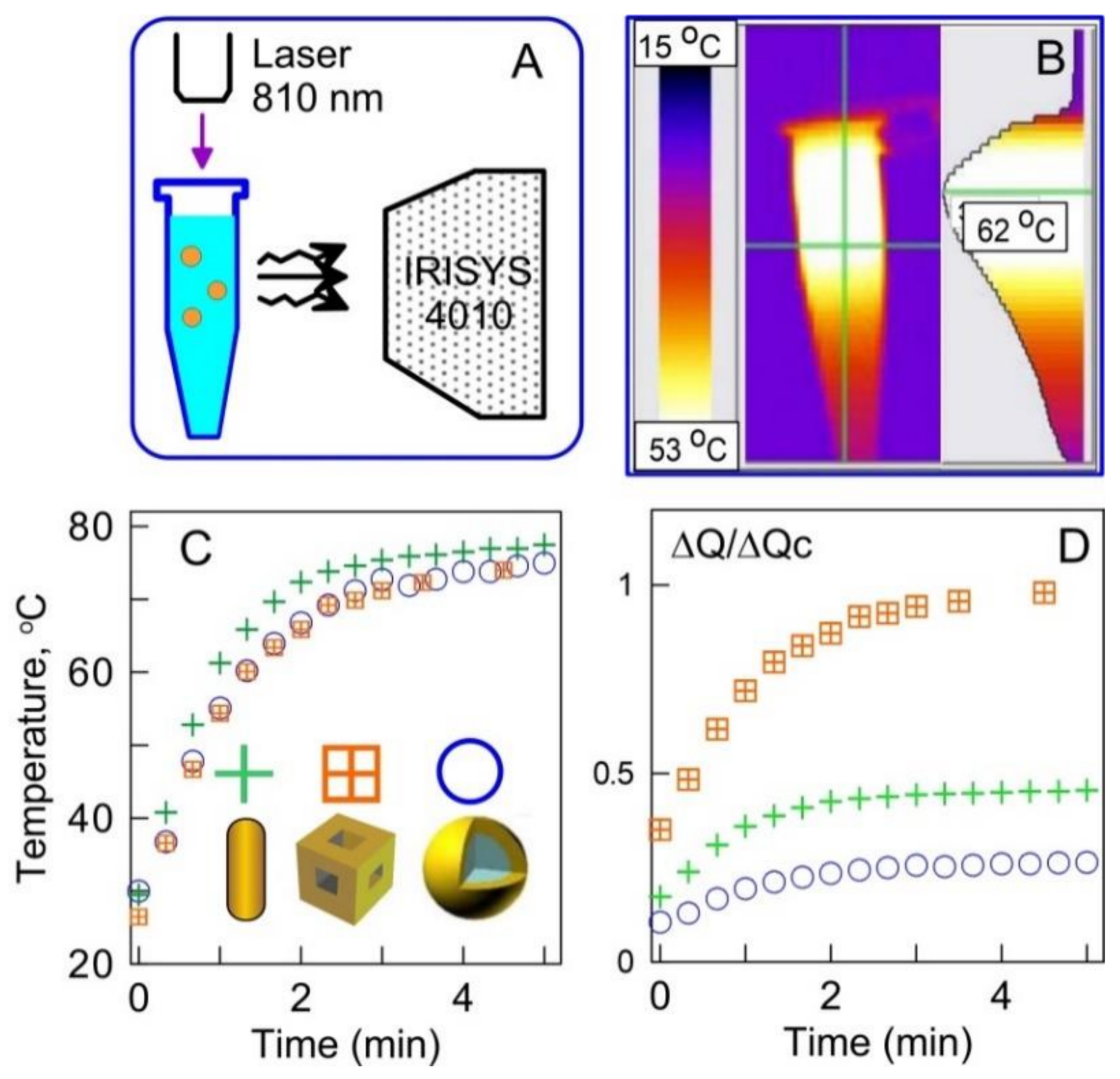
(**A**). Scheme of thermographic measurements with a diode laser at 810 nm and the particle suspensions placed in an Eppendorf tube. (**B**) The temperature distribution was recorded by a thermal infrared camera IRSYS 4010. (**C**) The kinetics of suspension heating for the three nanoparticle types. (**D**) The time dependences of heat production per weight unit of the metal normalized to the maximal value for the AuNCGs.

In our work [61], we compared the PPT efficiency of Si_2_O/Au nanoshells, AuNCGs, and AuNRs (Figure 8). The optical densities at 810 nm were adjusted to be equal for all three samples. The temperature distribution (Figure 8B) demonstrates the maximal heating in a suspension depth between the upper and bottom parts. This observation is explained by the interplay between the intensity decrease with deeper penetration and the phototransduction of light to heat. It follows from Figure 8C that all three samples demonstrate similar heating kinetics. However, the photothermal efficiency per unit gold mass is highest for AuNCGs, then for AuNRs, and finally for AuNSHs. Our conclusion about the superior efficiency of AuNRs compared to nanoshells agrees with data by von Maltzan et al. [118]. On the other hand, Rengan et al. [119] demonstrated excellent PTT efficiency of AuNCGs compared to conventional nanoshells.

Pattani and Tunnell [120] compared the heat generation and photothermal efficiency AuNSHs and AuNRs using an advanced measurement methodology and processing the data in terms of the Roper et al. approach [117]. Because of the larger size of nanoshells, their absorption cross-section was higher than that for AuNRs. Therefore, at an equal number concentration, the nanoshells produce more intensive heating per particle. However, the overall efficiency of PPT converting light into heat was twice as efficient for Au nanorods compared to Au nanoshells.

The shape effects of gold nanoparticles in photothermal cancer therapy were recently studied by Yang et al. [121]. Three nanoparticle types (AuNRs, AuNSTs, AuNSHs) were fabricated and functionalized with mPEG-SH to evaluate and compare PPT and antitumor efficiency. At 810 nm laser irradiation, AuNSTs showed the best performance. This result agrees with our observation of optimal optoporation efficiency of AuNST monolayers with growing cells [122].

Four PEG-functionalized nanoparticles (Au nanospheres, nanorods, nanocages, and nanoflowers) were recently tested as PPT agents [123]. The evaluated cellular uptake efficiency decreased in the following row: nanoflowers >nanospheres >nanocages >nanorods. Because the Au nanospheres have small NIR absorption, only three particle types can be used for NIR PPT. Among all nanoparticles tested, the Au nanoflowers showed the best PPT performance.

Robinson et al. [124] compared Au nanorods and nanocages as photothermal agents for the therapy of prostate tumors. It was found that Au nanocages demonstrate comparable PPT efficiency at 18 times fewer nanoparticles or half the total Au mass. Additionally, the biodistribution and excretion of nanocages were found to be more optimal compared to nanorods. This particular study points to the importance of further comparison of both nanoparticle types as applied to other tumor models. For example, a comparative study by Wang et al. [125] of three Au nanostructures (nanohexapods, nanorods, and nanocages) did not confirm any superior photothermal properties of nanocages. By contrast, the authors concluded that the most efficient PPT agents are nanohexapods that demonstrate better biodistribution, cellular uptake, and heating. Note that these results were reported for breast rather than the prostate tumor. Finally, Feng et al. [126] compared PTT and PDT efficiencies for Au nanorods, nanoshells, and nanocages under identical energy conditions and identical PR at 808 nm. The fabricated NPs demonstrated similar PPT properties but different PDT efficiency, highest for nanocages and lowest for nanorods. By comparing all in vitro and in vivo data, the authors suggested nanocages as promising candidates for combined PPT and PDT therapy. Thus, the existing comparative data are not convincing, and further studies are needed in the field.

### 2.6. Fabrication of Plasmonic Nanoparticles by Pulsed Laser Ablation in Liquid (PLAL)

**Figure 9:**
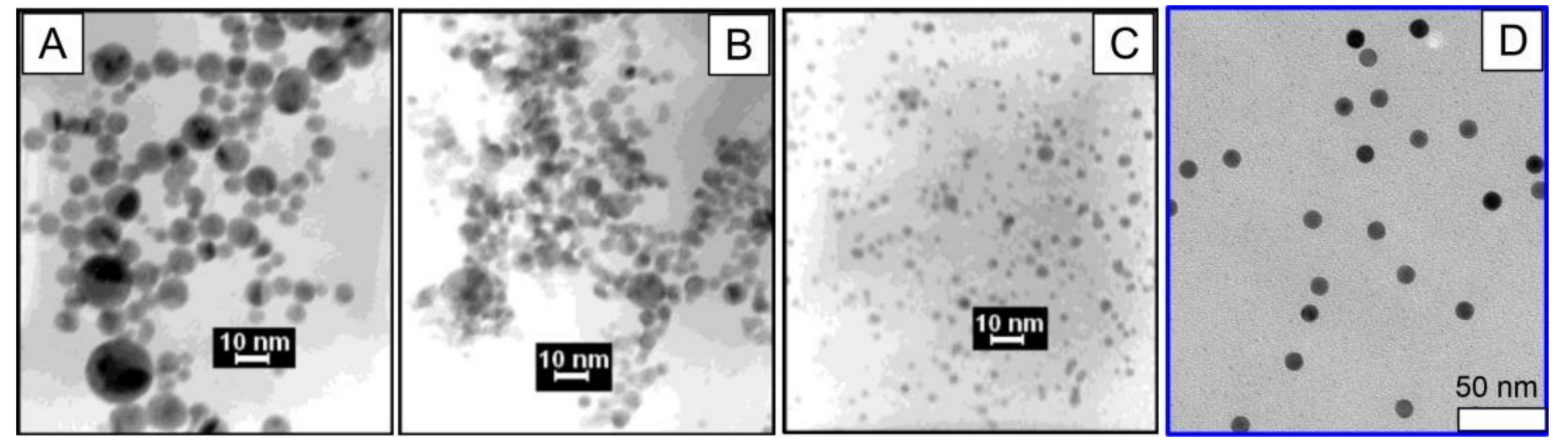
TEM images of Au nanoparticles prepared by femtosecond laser ablation in 0.1 mM (**A**), 1 mM (**B**), and 10 mM (**C**) *β*-CD. The average sizes are 10 ± 6.7 (**A**), 5.5 ± 3.7 (**B**), and 2.5 ± 1.5 nm (**C**). Copyright 2021 by American Chemical Society. Reprinted with permission from Ref. [128]. Panel (**D**) shows 10 nm particles synthesized by the method [129]. The scale bars are 10 nm (**A**–**C**), and 50 nm (**D**).

In 1993, Neddersen, Chumanov, and Cotton introduced a laser ablation method to prepare stable Ag, Au, Pt, Pd, and Cu colloids in water and organic solvents [127]. In this method, abbreviated as PLAL (pulse laser ablation in liquid) the metal in a solvent is irradiated with a pulsed Nd:YAG nanosecond laser for a while to prepare a stable colloid that is free from chemical reagents or ions associated with their surface. The size distribution of particles is controlled by varying the time of ablation and the pulse energy. To make the particle-size distribution narrower, Kabashin et al. [128] used a femtosecond PLAL technique for solutions containing cylodextrins (CDs). With an increase in CD concentration from 0.0001 to 0.01 M, the average particle size decreased from ~10 to 3 nm and FWHM decreased from 7–9 to 2–4 nm. However, even with the made improvements, the quality of particles and their size-distributions (Figure 9A–C) were not satisfactory as compared, for example, with 10 nm Au nanospheres fabricated in the Nanotechnology Lab (IBPPM RAS) by the method [129] (Figure 9D).

**Figure 10:**
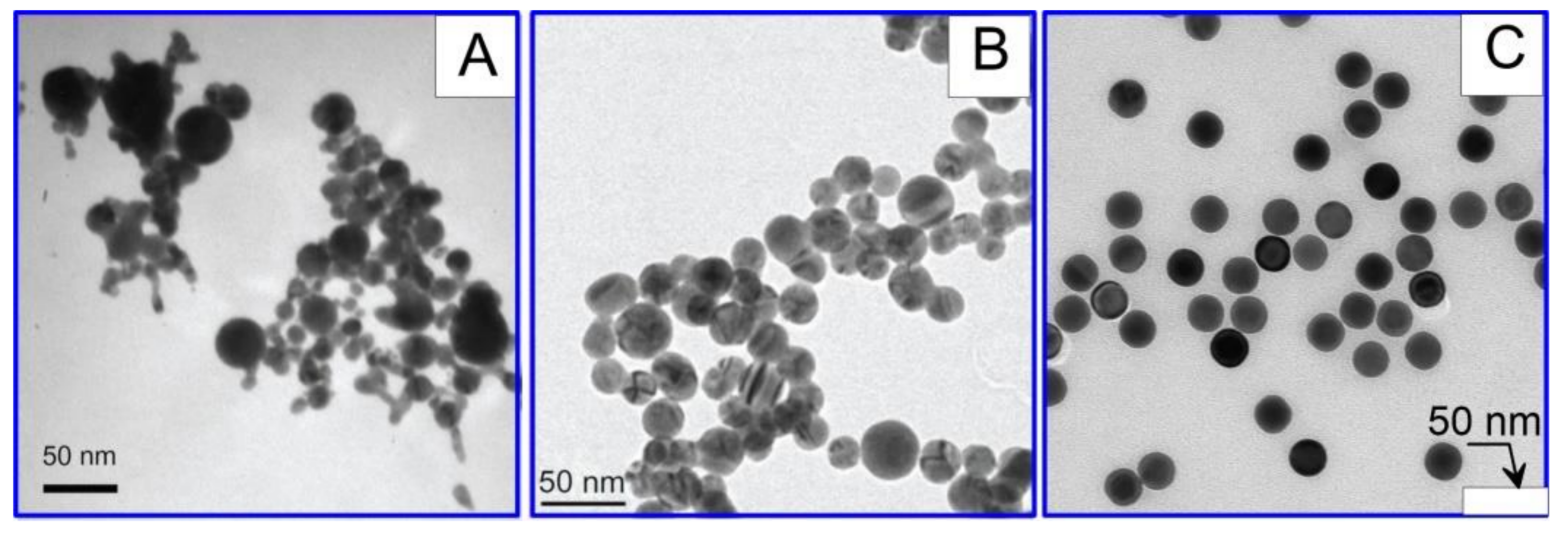
TEM images of AuNPs obtained after the first ((**A**), 55 ± 34 nm) and second ((**B**), 21 ± 6 nm) steps of ablation. Adapted from Ref. [131]. For comparison, panel (**C**) shows monodisperse spherical AuNPs (22.9 ± 0.4 nm) obtained by the method [129]. The scale bars are 50 nm.

Similar polydisperse samples were obtained in the study of surface chemistry of particles fabricated by fs PLAL technology [130]. To improve the particle quality, Besner et al. [131] suggested a two-step fs PLAL technique for obtaining AuNPs in deionized water. At the first step, the polydisperse ten-nanometer particles were fabricated, and then self-modification of the femtosecond laser pulse into a white-light supercontinuum was applied to finally obtain small AuNPs with reduced dispersion of size (Figure 10). Nevertheless, the wet-chemical method [129] gives monodisperse Au spheres with a much lower relative size dispersion of 1.7% as compared to the two-step femtosecond PLAL (28.5%). Similar results were reported for the fabrication of AuNPs by femtosecond-laser ablation and fragmentation in water [132]. In particular, the final fabricated particles had an average size 24 ± 5 nm. These particles were stable within two months, whereas other samples were prone to aggregation.

Despite numerous studies, the underlying physical mechanisms of PLAL are still debated [133,134]. Barcikowski and coworkers [135] found that PLAL of AuNPs is a one-pulse and one-step process producing unimodal particles with diameters less than 10 nm when the pulse peak power exceeds 1.6 × 10^12^ W/m^2^ and the educt particles are larger than 13.4 nm. They also found a correlation between the number of irradiation pulses (from 1 to 4) and the particle charge and surface chemistry. Recently, Bongiovanni et al. [134] used in situ electron microscopy to demonstrate the femtosecond laser fragmentation of AuNPs in water. They concluded that PLAL is a single-particle process involving Coulomb fission, which occurs as the femtosecond laser pulses ionize and melt the gold nanoparticle. This results in the ejection of highly charged progeny droplets. Subsequent events involve Coulomb fission accompanied by solution-mediated etching and growth processes. Thus, the fragmentation scenario produces complex PLAL patterns that rapidly fluctuate under further laser irradiation.

Braguer et al. [135] applied the method [133] for the fabrication of stable and monodisperse Au-NPs in water, PEG and, dextran solutions. The dextran-stabilized particles were found to be promising as drug carriers. An in vivo study on the safety, biodistribution, and pharmacokinetics of PLAL particles was reported in Ref. [136]. The PLAL method was also applied to the fabrication of Si@Au Core-Satellite Nanocomposites [137]. Khaniabadi et al. [138] showed that the low-energy X-ray dosage, together with PLAL treatment, results in a reduction in AuNP size, blue shifting of the PR wavelength, and some increase in negative zeta potential.

The use of PLAL needs ultrashort-pulsed lasers with high powers and megahertz-repetition rates which became available recently. However, there are problems with accurate optical laser pulse delivery on the metal target. Specifically, full lateral pulse separation is necessary to avoid a reduction in nanoparticle yield caused by pulse shielding. This point has been addressed in a recent report by the Barcikowski group [139].

**Figure 11:**
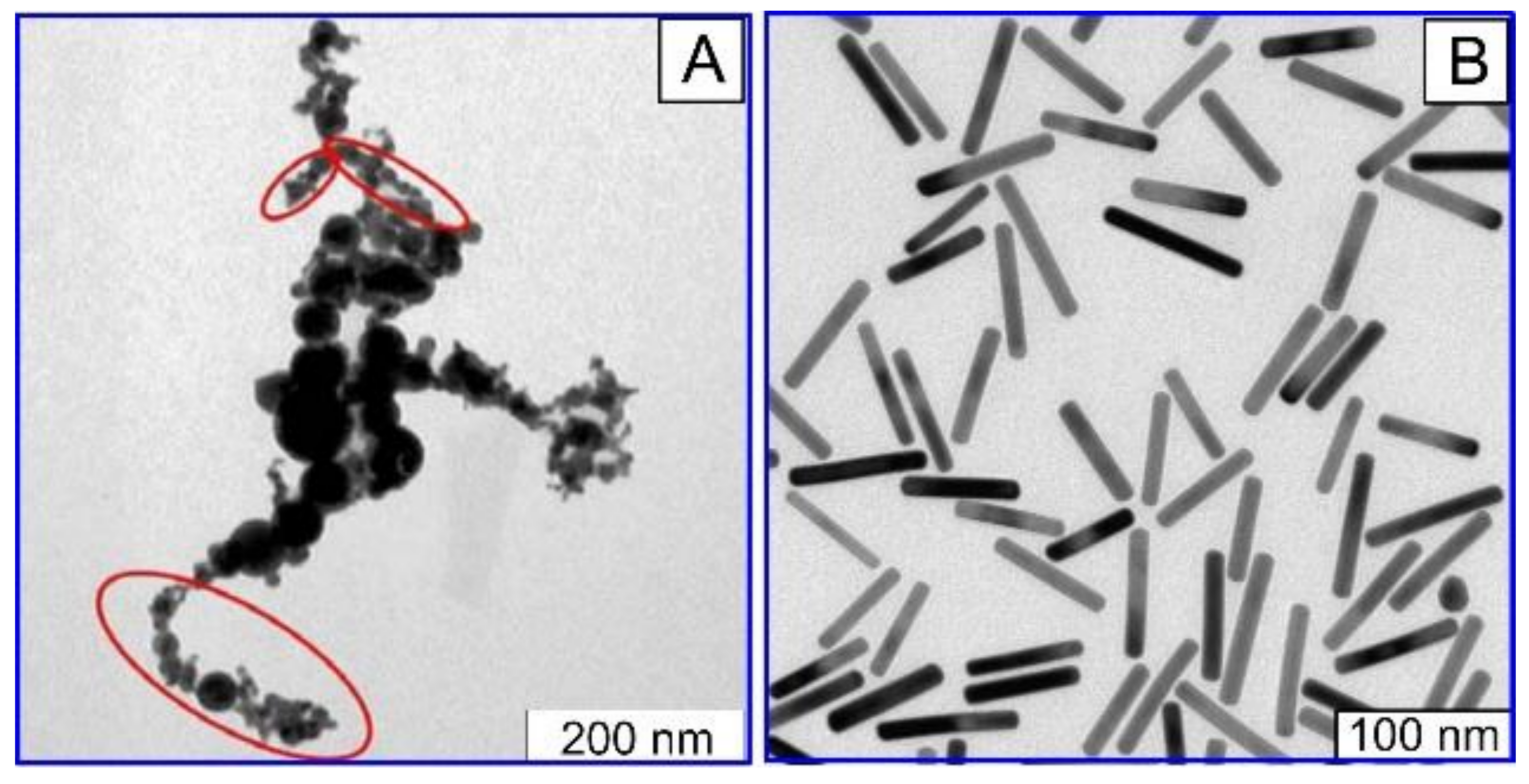
TEM image of chain-like elongated particles obtained after laser ablation in water (**A**). Adapted from Ref. [146]. For comparison, an ideally cigar-like shape of Au nanorods (Lab of Nanobiotechnology, IBPPM RAS) is demonstrated in panel (**B**).

In summary, we conclude that PLAL technique as applied to AuNPs is of limited possibilities for the precise control for the size, shape, and structure of particles as compared to existing well-established wet-chemical technologies. For example, the Shafeev group published several reports on the ablation of gold under laser irradiation [140,141,142,143,144,145], including reports on the formation of “elongated” Au nanoparticles under laser irradiation [146,147,148]. However, a close inspection of TEM images reveals the formation of complex nanostructures with some chain-like fragments (Figure 11A) or particles of a random shape [146] rather than distinct elongated single particles such as spheroids or rods.

Therefore, in fact, PLAL technique cannot be considered a versatile tool for shape control as compared to wet-chemical protocols (Figure 11B). Nevertheless, there are some specific topics where PLAL has promising potential. For example, the current fabrication of Si quantum dots involves cumbersome mechanical fragmentation and multiple separations. In this area, the laser ablation method [149] seems to be more effective compared to existing fragmentation-separation technologies.

## 3. The Nanoparticle Application for Antitumor Therapy

The application of nanoparticles as thermosensitizers for various types of hyperthermia has become a new trend in antitumor therapy. In oncology, electromagnetic radiation of different wavelengths, from radiofrequency to microwaves and ultrasonic waves, is used as heat sources for hyperthermia [150,151,152]. Hyperthermia is usually defined as the heating of a tissue to a temperature of 41–47 °C, less commonly temperatures in the range of 50–60 °C, for several minutes, which causes irreversible cellular damage through the appearance of disruptions in cell membranes and denaturation of proteins [153]. Tumor cells are more susceptible to hyperthermic effects than healthy cells because of their higher metabolic rate [154].

The laser application for hyperthermia makes it possible to obtain managed thermal damage of tumor tissue. However, the small spatial selectivity of tumor tissue heating remains a problem of laser hyperthermia [155,156]. The development of innovative nanoparticle-based technologies to improve the selectivity of laser heating is intensively pursued, and various types of plasmon resonance nanoparticles are used for this purpose, as follows: nanospheres [157] nanoshells [158], nanorods [159,160], nanocages [161,162]. In the current literature, plasmonic photothermal therapy is referred to by the acronym PPT [163].

The controlled synthesis of nanostructures allows adjusting the plasmon resonance of nanoparticles in a specific wavelength spectrum [164,165]. If the nanoparticles accumulate in the tumor tissue, the laser irradiation with a particular wavelength close to the plasmon resonance of gold nanoparticles causes thermolysis of tumor cells, while heating and damaging the surrounding healthy tissues does not occur [166,167]. For deeper tissue penetration of laser radiation, the tuning of the plasmon resonance of gold nanoparticles must be performed at the following wavelengths: 750–1100 nm, the so-called “therapeutic window of transparency” of tissues [168].

The passive and active delivery techniques are used to maximize the nanoparticle accumulation in the tumor tissue. Passive delivery is caused by the nanoparticle accumulation in the tumor tissue due to the effect of enhanced permeability and retention (“EPR effect”) characteristic of tumor vessels [169,170]. Disordered architectonics, arteriovenous shunts, changes in the shape and structure of endothelial cells, and enlarged pores in tumor vessels all make tumor vessels “perforated” [171,172]. Lack of functional lymphatic vessels in the tumor also reduces the elimination rate of nanoparticles from the tumor.

The effective and safe use of anticancer plasmonic photothermal therapy requires solving a number of problems related to the choice of the optimal method of nanoparticle delivery, reducing the nanoparticle accumulation in healthy tissues, developing methods to visualize their accumulation in the tumor, and optimizing therapy protocols [173,174,175]. The increase in nanoparticle accumulation in the tumor may be achieved by changing the size, shape, and coating of nanoparticles [176,177].

Thus, it was found that the use of polyethylene glycol (PEG) for nanoparticle coating reduces their agglomeration and increases their circulation time in the bloodstream [178]. For active delivery of nanoparticles, modification of their surface with tumor-specific markers was offered that can selectively bind to the membranes of tumor cells [179,180]. In research by O′Neal et al. [181], gold nanoshells were used for plasmonic photothermal therapy in mice with transplanted intestinal carcinoma; 6 h after intravenous injection of nanoparticles, 808 nm infrared laser with 4 W/cm^2^ power density was used to irradiate the tumor for 3 min, the authors noted a marked effect of therapy. Gobin et al. [182] intravenously injected gold nanoshells with an absorption maximum at 750–850 nm to mice with transplanted intestinal carcinoma, as follows: 20 h after injection, 808 nm infrared laser with 4 W/cm^2^ power density to irradiate the tumor for 3 min. On day 12 after exposure, a two-fold decrease in tumor volume and increased life expectancy of mice after photothermal therapy were detected.

Dickerson et al. [159] made the comparison of intratumoral and intravenous injection of gold nanorods in mice with squamous cell skin carcinoma. The authors noted that the most significant tumor accumulation of nanoparticles was observed 24 h after intravenous injection of gold nanorods, then this time point was applied for following PPT treatments for intravenous injections of AuNRs. It was found that 808 nm laser irradiation for 10–15 min at 1.7–1.9 W/cm^2^ was required for marked tumor destruction and minimal damage to surrounding tissues. However, a more pronounced effect was observed after intratumoral injection of gold nanorods on day 13 after photothermal treatment: the tumor volume decreased by 57% compared to the original volume, and with their intravenous injection it decreased by 23%.

Von Maltzahn et al. [118] found that at the same concentrations and PEG-coating gold nanorods are heated by laser irradiation six times faster than gold nanoshells. The same authors determined that PEG-coated gold nanorods circulate in the blood longer than PEG-coated nanoshells after intravenous injection. By intravenous infusion of polyethylene glycol-coated GNRs at a dose of 20 mg/kg in mice with melanoma xenograft MDA-MB-435, complete resorption of the tumor after photothermal therapy was achieved. This effect was probably due to the small tumor size and high dose of gold nanorods.

**Figure 12:**
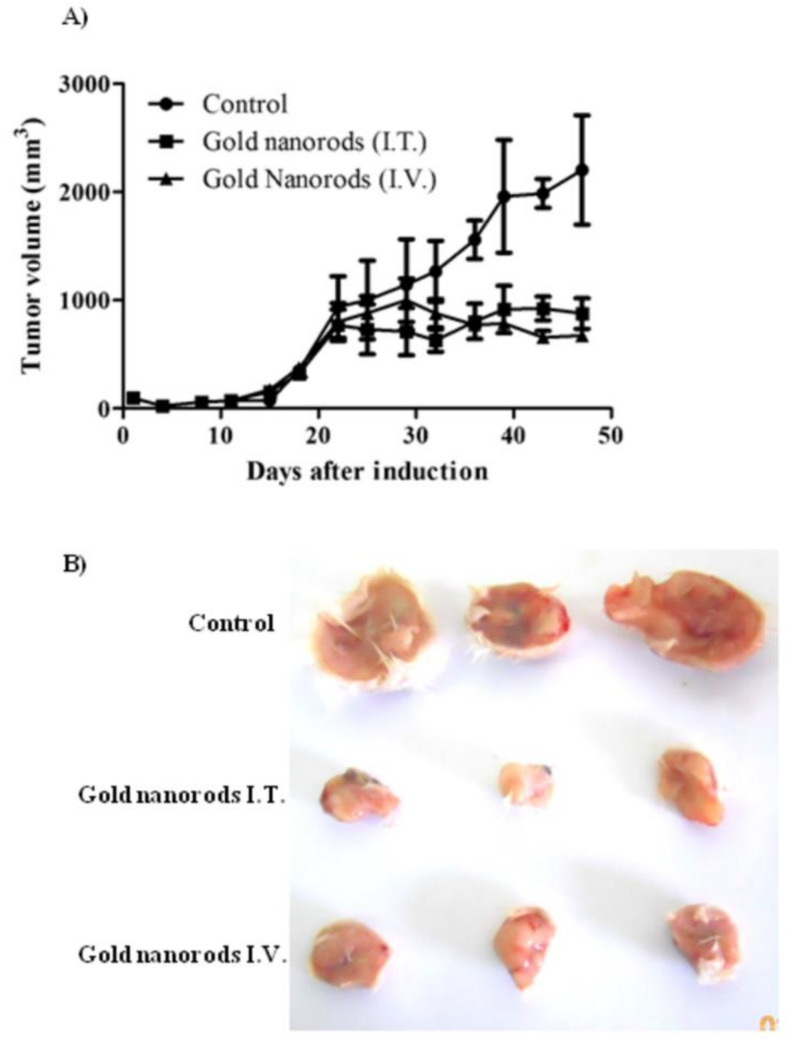
Antitumor activity of AuNRs coupled with laser-induced photo plasmonic thermal therapy in EACC solid tumor-bearing mice. EACC tumor-bearing mice were given gold NRs (1.5 mg/kg every three weeks) by I.V. (▲) and I.T. (■) administration compared to PBS-treated animals (●). Animals were exposed to a laser plasmonic beam (50 W/cm^2^ for 2 min) every week. Tumor size was measured every three days and plotted (**A**). Representative tumors are shown in panel (**B**). Data are presented as mean ± SEM (*n* = 10) Reprinted by CC BY license from Ref. [185].

In work by Chen et al. [183], a high dose of gold nanocages (9 × 10^12^ particles) was applied for intravenous injection in mice with transplanted glioma line U78 to increase their tumor accumulation. Photothermal therapy using an infrared laser with a low power density (about 0.7 W/cm^2^) resulted in a significant decrease in the metabolic activity of tumor tissues. The treatment was performed under the control of positron emission tomography. Tae and coworkers [184] have demonstrated the pronounced antitumor effect of PTT in nude mice bearing bilateral SCC7 tumors using intravenous GNRs loaded Pluronic F 68 nanocarriers. The significant suppression of the tumor growth was observed after irradiating the tumors with 808 nm laser for 4 min, 24 h after injection of GNRs. El-Sayed et al. [185] studied the multiple intravenous administration of polyethylene glycol-coated gold nanorods, with an axial ratio of 4.6 and an absorption maximum of 800 nm (1.5 mg/kg once every three weeks) in Balb C mice with Ehrlich carcinoma. Three days after nanoparticle injection, the most significant accumulation of gold in the tumors was detected. One week after each intravenous injection, the tumor was irradiated for 2 min with a diode laser with a power density of 50 W/cm^2^, and the tumor heating was up to 79 °C. However, inhibition of tumor growth was noted by the authors only from days 22 to 47 of observation (Figure 12).

In research by Sirotkina [186], gold nanorods of different sizes at a dose of 250 µg/kg (50/10 nm and 850 nm absorption maximum and 60/15 nm and 775 nm absorption maximum) were injected intravenously in CBA mice with cervical carcinoma. The nanoparticle accumulation in the tumor was visualized using OCT in dynamics for 7 h. At the highest accumulation of nanoparticles, the transplanted tumors were irradiated using a diode laser with a power density of 1.2–1.5 W/cm^2^, varying the laser power output to maintain the surface temperature of the transplanted tumor at 44–45 °C for 20 min. It is the case that 51% inhibition of the growth of tumors was observed one week after injection of 50/10 nm nanorods, after injection of 60/15 nm nanorods-the tumor growth inhibition rate was 72% [186]. Unfortunately, the small penetrating ability of OCT (2–3 mm) limits the use of OCT for visualization of nanoparticles accumulation in soft tissue tumors.

**Figure 13:**
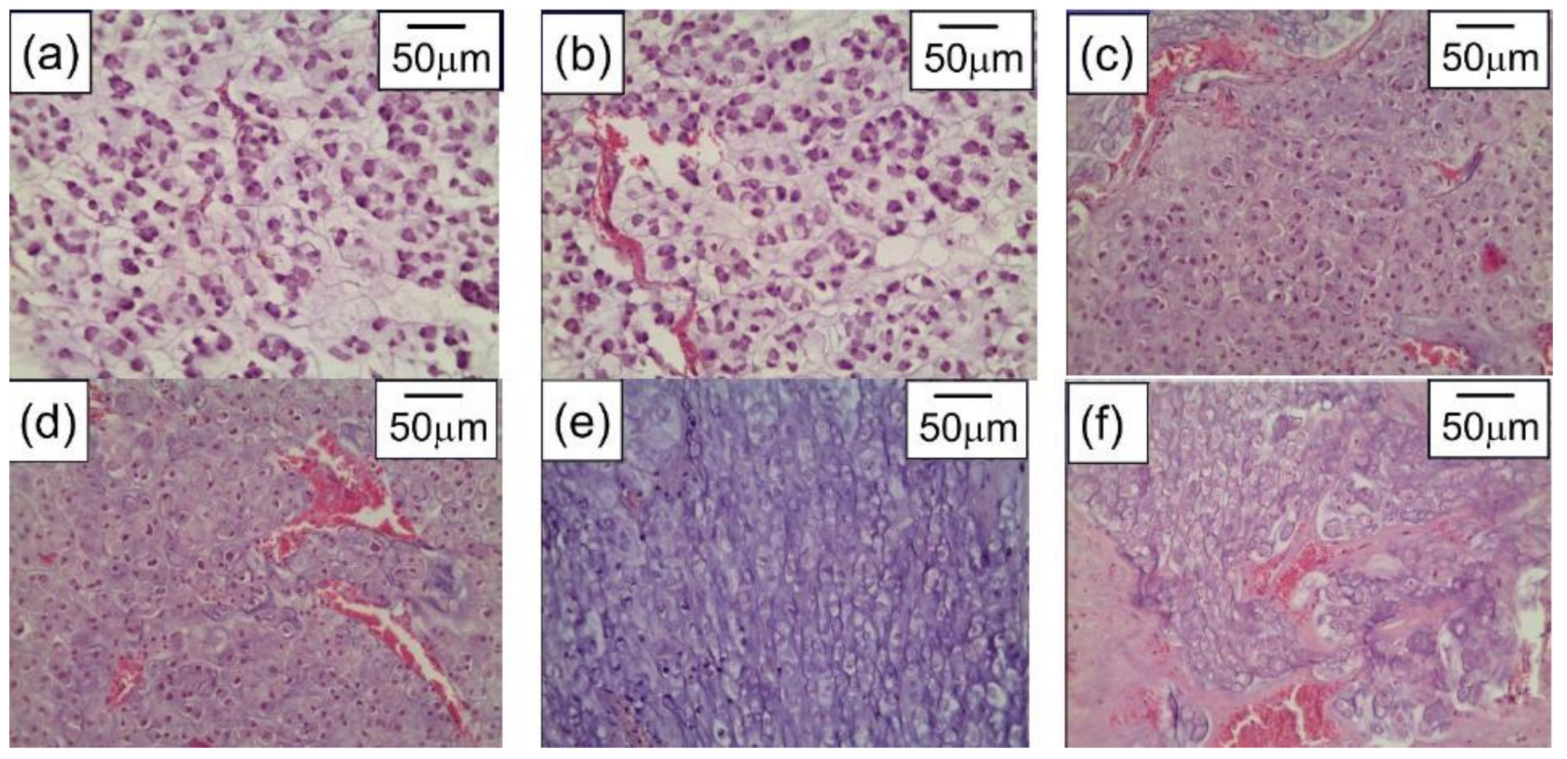
Cholangiocarcinoma PC-1–without any treatment (**a**), after only laser irradiation (**b**), after single IV administration of AuNRs and PPT (**c**), after double IV administration of AuNRs and PPT (**d**), after triple IV administration of AuNRs and PPT (**e**), and after intratumoral administration of AuNRs and PPT (**f**). H&E staining, magnification ×246.6. Copyright 2021 by Springer Nature. Reprinted with permission from Ref. [187].

In our research [187], the greatest accumulation of gold in tumor tissue in rats with transplanted cholangiocarcinoma was detected after triple repeated intravenous injections of gold nanorods. The subsequent tumor irradiation by 808 nm laser with 2.3 W/cm^2^ power density for 15 min was conducted 24 h after the last IV injection of AuNRs. The significant damaging effect of PPT after triple IV injection of gold nanorods showed up in expressed necrotic and degenerative changes in tumors (Figure 13).

However, if the accumulation of gold nanoparticles in the tumor tissue is insufficient, a slight temperature rise during laser hyperthermia can cause continued tumor growth. In addition, a significant problem is the optimal choice of time intervals between the intravenous injection of nanoparticles and the start of laser irradiation of the tumor to increase the efficiency of PPT therapy.

**Figure 14:**
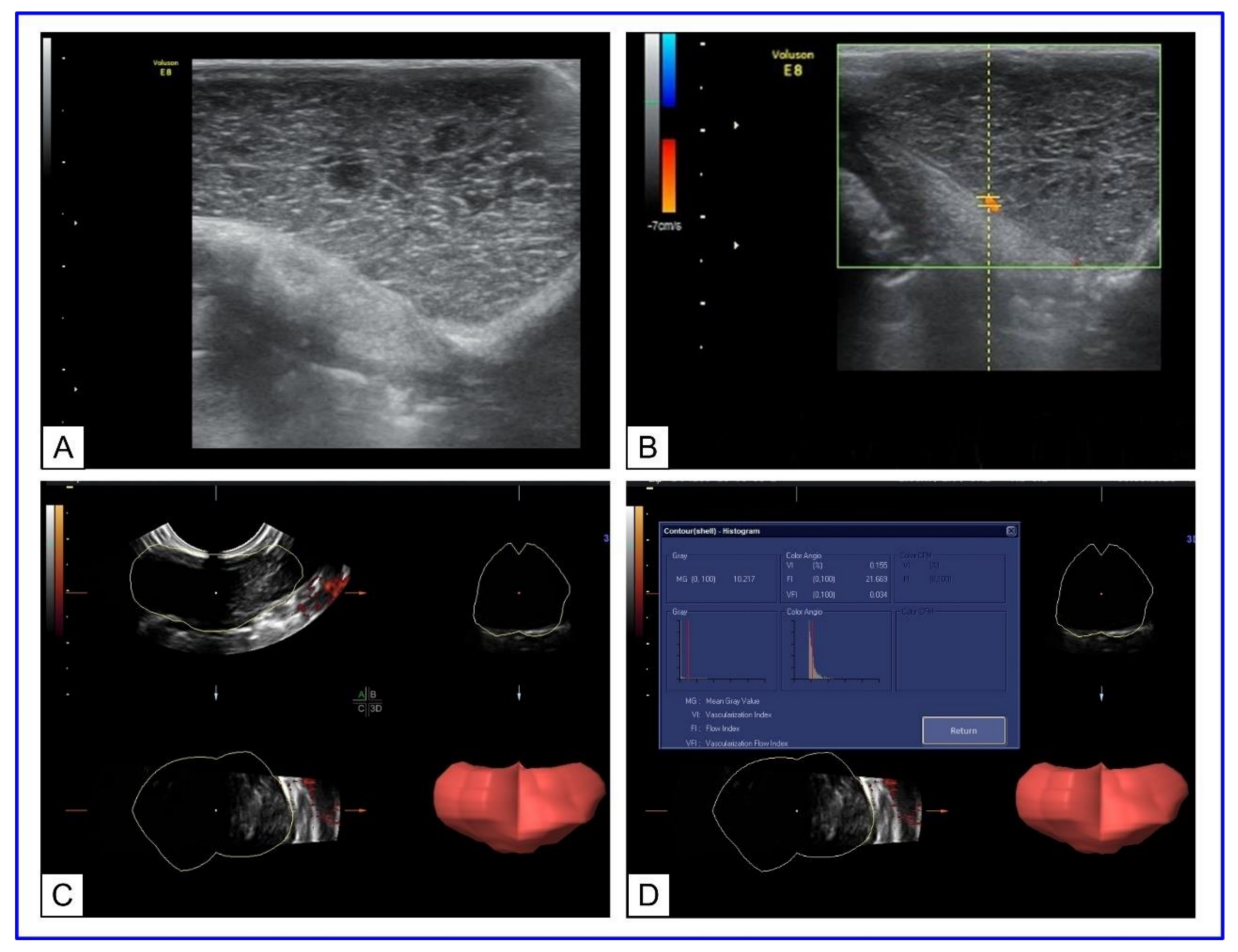
US Doppler imaging of rat tumors. (**A**) B-mode scanning; (**B**) 3D ultrasound mode. The lumen of the vessel is uniformly filled with color. (**C**) Three-dimensional (3D) power Doppler sonography in tumor-bearing rats. (**D**) Histogram analysis of vascularization indices. Copyright 2021 by John Wiley and Sons. Reprinted with permission from Ref. [188].

In our study [188], Doppler ultrasonography was conducted to describe transplanted tumors′ vascularity for optimal choice at the beginning of plasmonic photothermal therapy. AuNRs functionalized with thiolated polyethylene glycol (PEG) with an aspect ratio of 4.1 were used for multiple fractional intravenous administration in rats with transplanted cholangiocarcinoma PC-1. After the last injection of AuNRs, 808 nm laser irradiation of tumors, with 2.3 W/cm^2^ power density, was performed for 15 min by NIR diode laser LS-2-N-808-10000. The significant damage of tumor tissue with retardation of the tumor growth was observed after PPT treatment. The preliminary Doppler ultrasound allows to evaluate tumor vascularization and predict the efficiency of PPT depending on the degree of tumor vascularization (Figure 14).

A promising method of anticancer treatment is photodynamic therapy based on the use of photosensitizers [189]. After exposure to laser radiation with a certain wavelength, the photosensitizer goes to an excited state, and the formation of free radicals occurs, causing necrosis of tumor cells [190]. However, the major challenges of photodynamic therapy remain the achievement of the necessary accumulation of the photosensitizer in the tumor, the low penetrability of red laser radiation, insufficient oxygenation of many tumors, and slow biodegradation of photosensitizers [191,192]. The most commonly used photosensitizers are based on porphyrin [193,194], including Photosan^®^ in Germany, Photofrin^®^ in the USA and Canada, Alasens^®^ and Photosens^®^ in Russia. The use of metallic nanoparticles for conjugation with photosensitizers can develop combined methods of tumor therapy. The transition to the combined treatment of tumors using several alternative methods is a modern trend in experimental and clinical oncology [195,196]. A promising area of research is using a combination of phototherapy methods, namely, photothermal and photodynamic treatment for antitumor therapy. The use of laser radiation in both methods, acceleration of photochemical reactions during hyperthermia, synergistic effects on tumor blood vessels, time-difference of the therapeutic effects of PPT and PDT, increased thermosensitivity of cells in hypoxic conditions caused by the photochemical reaction-determine the effectiveness of combined therapy method [197,198,199]. Compared with conventional chemotherapy, combined phototherapy has significant advantages such as low invasiveness, high spatiotemporal selectivity, and rapid post-operation recovery [200,201].

Therefore, many research groups have proposed new treatment models that are based on the combination of PDT and PTT, using various types of nanoparticles and photosensitizers.

In 2014, Chen′s group proposed photosensitizer (Ce6)-loaded micelles integrated with cyanine dye for combined PTT/PDT treatment in mice with transplanted 4T1 tumors [202]. 24 h after intravenous injection of nanocomposites, the tumors suffered from various laser irradiation treatments, including PPT (785 nm, 5 min, 1.0 W/cm^2^), PDT (660 nm, 10 min, 1.0 W/cm^2^), combined PPT and PDT treatment (PTT/PDT), and reverse sequence of initial PDT and subsequent PTT treatment (PDT/PTT). The most pronounced tumor inhibition effect (~90%) was observed under sequential PTT/PDT treatments.

Zheng′s group proposed internalized RGD-modified indocyanine green (ICG) liposomes for PTT/PDT treatment of mice breast tumors 4T1. After 24 h intravenous injection of liposomes, the tumors of mice were irradiated by the 808 nm laser at a power density of 1.0 W/cm^2^ for 10 min. The tumor growth was significantly inhibited (up to ~98%), achieving almost complete tumor regression [203]. In 2018, Shen′s group reported that the assembly of iron oxide carbon dot NPs conjugated with black phosphorus quantum dots exhibited significant tumor-inhibition efficacy due to the synergistic PTT and PDT treatment via a near-infrared laser [204].

In combined photothermal and photodynamic therapy, several studies use nanocomposites with a gold core, which determines the heating during PPT, and a silicon shell containing encapsulated photosensitizer molecules responsible for the photochemical reaction. The common photosensitizers used in conjunction with the plasmonic NPs include phthalocyanines [205], toluidine blue, indocyanine green, and porphyrin derivatives [206]. Low toxicity of such nanocomposites was noted in a number of in vitro studies [207,208,209], a large number of articles are devoted to their in vivo application [210,211,212,213]. Jang B. et al. [210] developed gold nanorods conjugated with aluminum phthalocyanine tetrasulfonate (AuNR−AlPcS4). After intravenous injection of AuNR−AlPcS4 complex, tumor growth reduced by 79% with photodynamic therapy (PDT) alone and by 95% with dual photothermal therapy (PTT) and PDT in tumor-bearing mice. Wang S. et al. [211] reported a combined PDT/PTT treatment, in which MDA-MB-435 tumor-bearing mice received an intratumoral injection of gold nanostars conjugated with chlorin e6 (GNS-PEG-Ce6), followed by 6 min of 671 nm laser irradiation at 1.0 W/cm^2^ at four h post-injection. The early phase PDT effect was coordinated with the late-phase photothermal effect, indicating the high heat conversion and remarkable anticancer efficiency of Ce6-conjugated gold nanostars.

**Figure 15:**
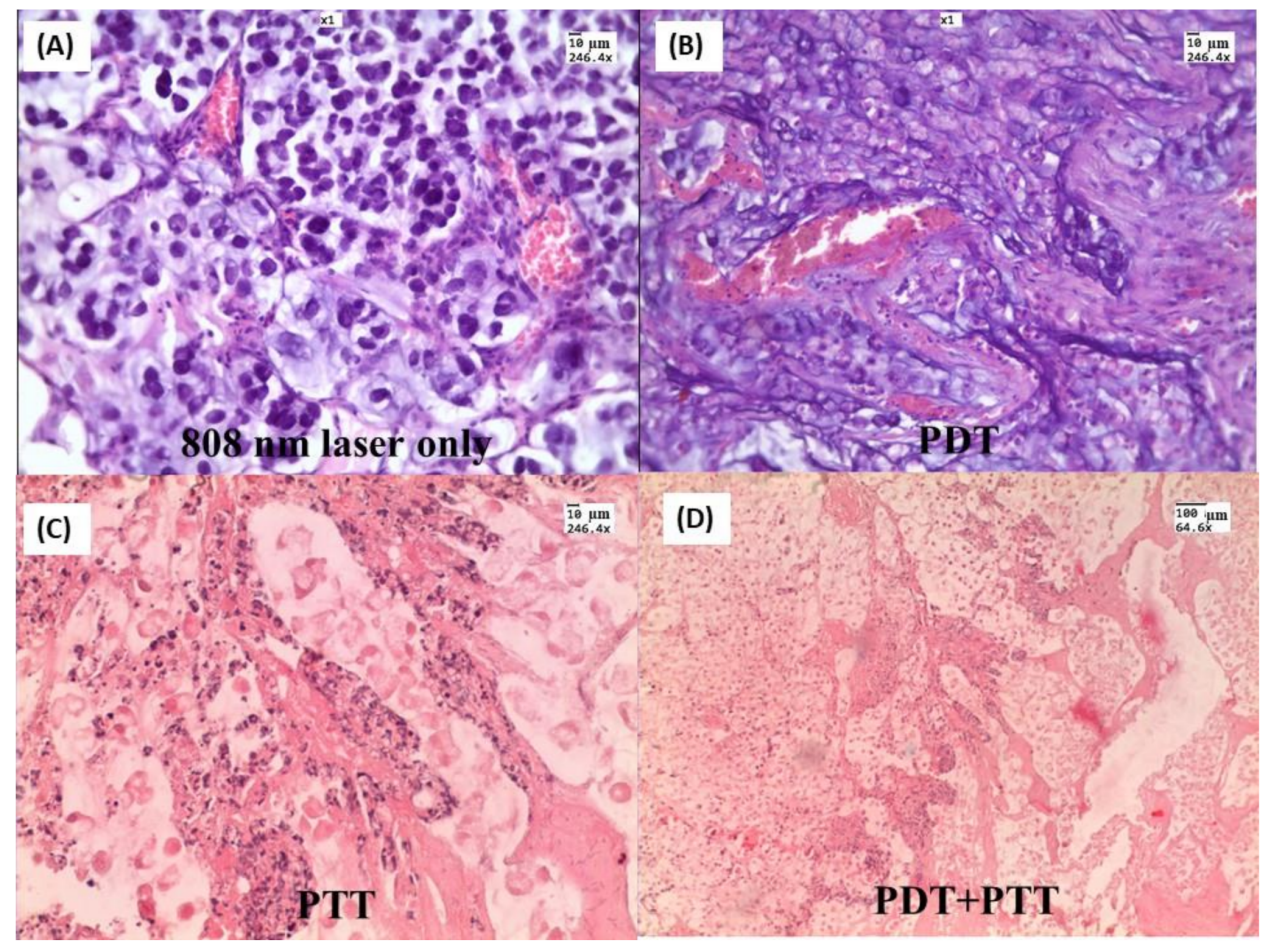
H&E-stained tumor slices after different treatments: (**A**) Laser 808 nm 2.3 W/cm^2^ treated only; (**B**) NCs injected and 633 nm 160 mW/cm^2^ treated; (**C**) NCs injected and 808 nm 2.3 W/cm^2^ treated; (**D**) NCs injected and simultaneously irradiated with two lasers. Tumor tissue specimens were obtained three days after treatment. Copyright 2021 by Springer Nature. Reprinted with permission from Ref. [212].

In our work [212], nanocomposites (NCs), consisting of a gold nanorod core and a mesoporous silica shell doped with hematoporphyrin, have been fabricated for combining photothermal and photodynamic therapy (PDT + PTT) in rats with transplanted cholangiocarcinoma. NCs were directly injected into tumors and irradiated simultaneously with 633 nm and 808 nm lasers. In the comparison group with only PDT, weak changes in tissue histology and a moderate 20% decrease in the tumor volume were observed. In contrast, the combined PDT + PTT treatment resulted in large-area tumor necrosis (Figure 15) and dramatically decreased tumor volume. Zang S. et al. [213] proposed silica-coated gold nanorods conjugated with 4-carboxyphenyl porphyrin (AuNR@SiO_2_-TCPP) for combined photothermal and photodynamic therapy in mice with A549 xenograft tumors. The tumor growth in mice receiving AuNR@SiO_2_-TCPP with subsequent 660 and 808 nm irradiations was significantly inhibited, and the average tumor volume was decreased almost ten-fold compared to the control group.

Compared with other functional nanomaterials for combined PDT/PPT therapy, the gold NPs demonstrated comparable biocompatibility and tumor inhibition performance, implying a promising potential for cancer therapeutics. However, the common combination of PDT and PTT needs to be activated by two separate lasers with different excitation wavelengths, which results in the prolongation of treatment time and the complication of the therapeutic process. Therefore, it is necessary to develop nanocomposites irradiated by a single wavelength laser to generate hyperthermia and ROS, triggering PPT and PDT simultaneously.

In recent work, Liu et al. [214] designed nanocomposites based on gold nanorods surface-functionalized with chlorin e6-C-15-ethyl ester (HB) and tumor-targeting peptide cyclic RGD (cRGD) to develop HB-AuNRs@cRGD for single NIR laser-induced targeted PDT/PTT. After intravenous administration in BALB/c female nude mice with ECA109 esophageal cancer model, the HB-AuNRs@cRGD could be preferentially accumulated within tumor sites and rapidly internalized by cancer cells. The pronounced damage of tumor cells with nuclear membrane fragmentation (karyorrhexis) and nuclei shrinkage with pyknosis was observed from tumor tissues in the HB-AuNRs@cRGD group with 660 nm laser irradiation, which could induce intensive necrosis or apoptosis, the tumor inhibition rate was approximately 77.04% [215].

Although phototherapy based on a combination of PDT/PPT treatments has been widely studied, many projects are still in the early preclinical stage, and there are still many deficiencies that need to be improved. Several unresolved problems are associated with the use of nanostructures for combined therapy, including those related to the choice of optimal doses and methods of nanocomposite administration, laser radiation protocols, and the most effective combinations of therapeutic effects. In addition, the safety, biocompatibility, and efficient targeting of tumor cells by PTT and PDT based on nanoparticles must be considered, and the clinical applicability for the treatment of various diseases must be further investigated.

## 4. Evaluation of Optical Properties of Tumors and the Propagation of Laser Radiation and Heat in Models Sensitized with AuNPs

Modeling the conversion of energy from light to heat by nanoparticles and the heating of the tissue surrounding the particles, as well as its kinetics, is important for the development of PPT. The efficiency of such energy conversion of gold and some other metallic nanoparticles under optical irradiation was evaluated using Monte Carlo modeling, described in Refs. [215,216]. The modeling of temperature fields in tumors with embedded AuNPs during PPT and model experiments with intratumorally injected nanoparticles have shown that the heating of nanoparticles and surrounding tissues is a fast enough process. For example, at the laser pulse duration of 2 ns and the intensity of 5 mW/cm^2^ at the plasmon resonant wavelength, the temperature increment in the gold nanoparticle reaches about 100 °C [217]. In Ref. [218], rapid heating of nanoparticles (picoseconds), which caused the initial peak of the photothermal signal, was detected. The increase in laser intensity led to the formation of nanobubbles around the superheated nanoparticles. The method of selective cancer cell thermomechanical destruction during laser irradiation at the wavelength of plasmonic resonance was patented [219].

Traditionally, the transport of radiation in tissue can be modeled either on the basis of a diffusion approximation approach or on the basis of stochastic analysis that takes into account the statistical uncertainty of radiation propagation in tissue. The analytical approach in combination with the Arrhenius damage integral was used by Yakunin et al. [220] for accurate quantitative assessment of hyperthermia and biological effects in conditions when the exposure time varied from tens to hundreds of seconds, and the generated heat affected an area of tens to hundreds of nanometers near the nanoparticle. In Ref. [221], nanoparticles were embedded subcutaneously at a depth of 1 mm, and the maximum temperature at the spot center after continuous laser irradiation with the intensity of 10 W/cm^2^ during 30 s achieved 65 °C. At this temperature, for perfusion rates typical of the cutaneous covering, the perfusion affected the thermal regime only at times longer than 60 s [222].

In most models, uniform distribution of the nanoparticles in tumors after the intratumoral injection is assumed. Therefore, the tumor is modeled as one object with homogeneous optical properties. However, knowledge of the real distribution of nanoparticles in tumors allows for correcting heat transport modeling during PPT. Von Maltzahn et al. [118] used noninvasive X-ray computed tomography or ex vivo spectrometry to obtain AuNR biodistribution data in tumors after intratumoral or intravenous administration, respectively, and four-dimensional computational heat transport modeling to predict real photothermal heating.

The use of PPT in antimicrobial therapy is widely studied [223,224,225,226,227]. Results of the photothermal effect of AuNPs on microorganisms have shown to be dependent on the shape and optical properties of the nanoparticles and properties of laser radiation [228]. The formation of a local temperature field in suspensions of microorganisms with embedded AuNRs under irradiation with a NIR laser (power density of around 100 mW/cm^2^) was considered theoretically in Ref. [229]. It was found that AuNPs functionalized by human immunoglobulins IgA and IgG formed “clouds” around cells and induced temperature rise in the microscale zone.

Knowledge of the optical properties of tumors is beneficial for analyses and optimization of the parameters determining laser energy absorption. Many research teams are working in this direction. The optical properties of various tumors in a wide wavelength range have been reviewed in Ref. [230].

Typical parameters of laser irradiance at the surface during PPT are the following: 2–50 W/cm^2^ power density, 1–5 mm spot size, and a wide range of heating duration (from shorter than several minutes to longer than 15 min) [181,182,183,184,185,186,187,188,212,231]. These parameters are used for computational Monte Carlo simulation of laser irradiation in tumors. For example, a Monte Carlo algorithm was developed by Manuchehrabadi et al. [232] to simulate photon propagation (808 nm, 1.6 W/cm^2^) in a spherical tumor after intratumoral injection of AuNRs calculate the absorption of laser energy in the tumor and investigate the effect of absorption and scattering coefficients on the simulated heating.

The change of the penetration depth of the laser light (1064 nm) in a tissue model with three layers of skin, adipose tissue, and muscle, before and after application of glycerol, were investigated by Youn [233]. By substituting the absorption and scattering coefficient into a numerical simulation of the temperature distribution, it was found that the application of optical immersion agents during laser treatment can reduce heat generation on the skin surface and stimulate the tissue′s heating deeply. The simulation was confirmed by experimental data [233,234].

The thermal effect on tumor tissues at different depths can be assessed by measuring the optical parameters of irradiated and non-irradiated tissues. Until now, this problem remains insufficiently studied. The changes in the optical parameters of biological tissues under the influence of temperature have been presented in a lot of works, for example, for blood [235], brain [236,237], skin [238,239], prostate [240], liver [241,242], etc. However, changes in the optical parameters of tumors during PPT require detailed research.

It should be noted that the main accumulation of nanoparticles during intravenous injection occurs in the blood vessels of the tumor, which are localized mainly in the peripheral part of the tumor. The intratumoral injection also does not allow for a uniform distribution of particles inside the tumor. Thus, the heating of the tumor tissue occurs unevenly. To optimize the laser exposure, information about the heating of the inner layers of tumor tissue in vivo is required. In our works [243,244], optical properties of main layers of model cholangiocarcinoma in rats (capsule, top part, center, and bottom part) and also bordering layers of skin and subcutaneous tissue were evaluated in the spectral range 350–2200 nm before and after PPT. In these studies, AuNRs were injected intravenously or intratumorally with different doses, and IR diode laser (808 nm, 2.3 W/cm^2^) was used for irradiation. In Ref. [244], combined use of an immersion agent with low-intensity laser irradiation (1.5 W/cm^2^) for optical clearing of the skin before PPT was proposed. The effect of immersion agent (mixture of 70%-glycerol and 10%-dimethyl sulfoxide) on the optical parameters of the skin, subcutaneous tissue, and a model tumor in rats in vivo after hyperthermia caused by PPT was presented as a pilot result.

## 5. Combining PPT with Other Therapies to Achieve Synergic Efficiency

One of the most promising approaches is the integration of PPT with other techniques to achieve advanced synergic anticancer therapy. There are at least five possible ways for such integration: (1) PTT + PDT; PTT + chemotherapy (PTT + CHT); (3) PTT + immunotherapy (PTT + IMT); (4) PTT + gene therapy (PTT + GT); (5) PTT + radiotherapy (PTT + RT) [245]. An interesting PPT/PDT agent is pCo_3_O_4_ nanoplates. Because of the unique properties of Co_3_O_4_ NPs, their PTT/PDT and other functions are in ongoing examinations in many laboratories worldwide. In particular, Yuan et al. [246] reported multifunctional abilities of pCo_3_O_4_ NPs as photoacoustic/magnetic resonance contrast-imaging agents and as NIR-triggered PTT/PDT therapeutic nanoformulations. Furthermore, they also confirmed the suppression of the epithelial–mesenchymal transition (EMT) pathway by pCo_3_O_4_ NPs.

In recent years, several groups reported the unusual ability of AuNRs [247,248,249], AuNCGs [250], AuNSHs [251], and bipyramids [252] to generate singlet oxygen, thus enabling PDT treatment. Because of plasmonic absorption, all the above NPs would be suitable for combined PPT/PDT therapy. Unfortunately, there are only a few reports on the subject. Therefore, it is not clear whether this approach can find experimental confirmation. In any case, a close examination of reported ABDA oxidation plots clearly points to a low efficiency in ROS producing under irradiation of bare Au nanorods or bipyramids. According to our experimental observations, Au atomic nanoclusters can produce singlet oxygen under laser irradiation. However, the level of delivered singlet oxygen concentration is much lower than can be observed with common PDT photosensitizers. Therefore, including the common effective PDT agents such as chlorin e6 into therapeutic composites seems to be a more promising way. To summarize, in spite of attractive properties [253], the combined PPT/PDT approach can suffer from the limited NIR irradiation in deep tissue layers. In addition, the excitation wavelengths for PTT and PDT sensitizers can be different, thus reducing the efficiency of combined therapy. Therefore, further research is needed in this area.

According to the literature data [248,254] (still a few), the combined use of PTT and CHT can cause a number of positive effects: (1) accelerate the penetration of drugs into cancer cells; (2) reduce the side effects of CHT (3); remove cancer tissues more effectively; (4) reduce multidrug resistance. The most striking manifestation of the synergism of PTT and CHT is the direct enhancement of the cytotoxicity of well-known drugs used in oncology, such as cisplatin, carboplatin, cyclophosphamide, carmustine. When combined with PPT and an increase in temperature from 37 to 40 degrees, a linear increase in cytotoxicity was observed [245]. In a study by Urano et al. [255], a significant increase in cytotoxicity was observed with an increase in temperature in the range of 40.5–43 degrees. The ineffectiveness of PTT may be associated with the activation of heat shock proteins (HSPs), leading to an increase in the resistance of cancer cells to heat. Under conditions of a heterogeneous tumor and a strong attenuation of radiation in deep tissue layers, activation of HSPs can minimize the effect of PTT. On the other hand, the inclusion of several chemotherapy drugs in one nanoformulation can increase the synergistic effect of PTT and reduce the side effects of therapy.

Compared to radiation therapy and chemotherapy, IMT relies on the use of the patient′s immune system. Typically, the patient′s immune system is unable to respond with a protective response against tumor development because cancer cells reduce the stimulation of T lymphocytes [256]. When various immune cells, including dendritic cells and T cells, invade the lesions, the activation of this antigen-specific immune response is mainly suppressed due to the immunosuppressive effect of TME. The method of immunotherapy is based on the activation of immune cells capable of infiltrating a tumor by triggering an antigen-specific immune response, since cancer cells at the site of the tumor release specific antigens. Interestingly, PDT treatment of cancer can induce immunogenic call death [257]. Although there are several reports on the successful integration of PTT and IMT [245,258,259,260], the lack of clear understanding of all details of PPT-modulated immune response is an open question.

The use of GT in cancer therapy is based on triggering apoptosis of tumor cells, increasing the level of cytotoxic immune cytokines, and suppressing HSP expression. In addition, cellular uptake of DNA or RNA is usually tricky due to their degradation by enzymes. Delivery of DNA and RNA using photothermally inducible agents may solve this problem [261]. In addition, PTT can induce improved gene silencing due to enhanced endosomal escape of gene delivery vectors [262]. The main obstacles to in-clinic translation of PTT + GT technology are biocompatibility and toxicity concerns, together with a small volume of experimental data.

In contrast to optically induced PDT and PTT technologies, RT has no depth limitation to kill tumors. That is why computer tomography-guided RT is a powerful clinic technology [263]. However, tumor hypoxia is the main obstacle in RT applications at low linear-energy transfer, typical for many photon/electron beams, because the O_2_ concentration controls the DNA damage by reactive oxygen species. Nevertheless, recent reports [264,265] showed promising capabilities of integrated RT and PPT technologies.

## 6. Future Perspective and Limitations

Owing to significant progress in wet-chemical technologies, the researchers now have great possibilities in the fabrication of plasmonic NPs with almost any desired optical properties and surface modification. From a fundamental point of view, the absorption and scattering of light, the generation of heat, and its diffusion into the environmental medium are well established. On the other hand, many practical aspects of PPT, especially for in vivo models, are still far from complete understanding. As a result, the progress in the translation of laboratory PPT demonstrations in clinical practice is still unconvincing. Yet, there is an evident gap in our understanding of critical parameters determining the PPT efficiency of plasmonic NPs. Indeed, the above discussion in Section 2.5 clearly demonstrated the controversy in existing experimental reports and claimed conclusions. Clearly, there is an evident need for further studies aimed at the standardization of particle parameters, measuring procedures, and data processing. For example, the normalization of suspension by the particle numbers, the total metal mass, or the optical absorbance may result in a controversial conclusion.

The following vital point is a pretty poor translation of plasmonic NPs into clinical practice. This can be related to many reasons. One of the main problems is insufficient accumulation contrast of NPs in the targeted tissue. Initial hoping was the EPR effect in solid tumors, although the underlying EPR concept was debated in the literature [266]. To date, there is a consensus about the overestimated role of EPR in the prediction of NP accumulation and, as a result, the PPT efficiency [267].

The next concern is the biodistribution, toxicity, and long-term fate and clearance of inorganic particles (such as Au NPs) in the body [173]. Variations in the particle size, shape, structure, and surface chemistry of nanoparticles, together with specific features of cells and tumor models in vivo, make the toxicity problem to be dependent on too many parameters to be resolved in all details. Most encouraging preclinical studies were obtained with simple in vivo cellular models and subcutaneous xenograft murine models. Both systems are too far from actual cancer problems and clinical scenarios. Therefore, further development of PPT needs adequate in vivo and in vitro models, as different cell cultures and animals have different heat responses and thermoregulation.

One of the main obstacles of PPT is the strong absorption and scattering of light by cells and tissues in vivo. Therefore, we encounter two opposite demands: (1) to make the light irradiation as safe as possible for healthy organs and (2) to deliver the desired light power to targeted PPT sites. The possible solutions are the use of appropriate light waveguides and optical clearing techniques [268,269,270,271].

Finally, most previous cited works considered a simplified optical model of single-particle absorption and scattering approximation Equation (13). For more realistic multiscale modeling, one has to consider a more complicated optical approach which includes both the multiple scattering and absorption through the radiative transfer equation and the tumor heating by multiple sources, which are randomly distributed in the tumor. The work by Sokolovskaya et al. [272] exemplifies such multiscale modeling as applied to the subcutaneous tumor in skin illuminated by a laser. Silicon nanoparticles with diameters ~80 and 160 nm were used as a model for light-to-heat sources. One of the main conclusions is a nonlinear relationship between the particle concentration and the spatial distribution of temperature in the tumor because of the absorption and scattering shielding effect in any multiple scattering and absorbing medium. In particular, the author found an optimal mass–volume concentration 3 mg/mL. Two important notes are in order here. First, the absorption cross-section of silicon particles is several orders lower compared to that for gold nanoparticles. Specifically, *C_abs_* = 1 nm^2^ for Si particles and 1500 nm^2^ for Au particles with the same diameter of 30 nm. As a result, the volume concentration of Si particles needed to heat the tumor for 40 K was ~2 × 10^−3^ (5 mg/mL) at an extremely high intensity of the incident light 100–300 mW/cm^2^. By contrast, similar heating can be achieved with Au nanorod volume concentration 2 × 10^−5^ (0.4 mg/mL) at moderate incident light intensity 2 mW/cm^2^ [273].

## 7. Conclusions

In this review, we have summarized the existing theoretical models for approximate yet accurate simulations of optical cross-sections of plasmonic particles. For PPT applications, most important is accurate modeling of absorption cross-sections for particles of various shapes and structures. We have also provided a unified approach to the plasmonic tuning of different nanostructures for maximizing their absorption properties to NIR and SWIR biomedical optical windows. The current wet-chemical robust protocols to fabricate desired nanoparticles have been considered and exemplified by the most popular nanoparticle types. From a PPT point of view, the effective delivery of heat to targeted cells and organs needs an understanding of physical mechanisms underlying the conversion of light to particle heat followed by its transport to the external medium. We have reviewed basic models and results related to state-of-the-art plasmonic heating. In the second part of the review, we have considered recent experimental data on the applications of PPT and combined PPT + PDT to cancer therapy.

## Abbreviations

ABDA—9:10-anthracenediyl-bis(methylene) dimalonic acid; APTMS—3-aminopropyltrimethoxysilane; AuNCG—gold nanocage; AuNR—gold nanorod; AuNSH—gold nanoshell; AuNST—gold nanostar; CD—cylodextrin; CHT—chemotherapy; CTAB—cetyltrimethylammonium bromide; EG—ethylene glycol; GT—gene therapy; HSPs—heat shock proteins; IMT—Immunotherapy; NIR—near-infrared; NP—nanoparticle; PDT—photodynamic therapy; PEG—polyethylene glycol; mPEG-SH—mPEG-Acetamide Ethanethiol; PLAL—pulse laser ablation in liquid; PPT—plasmonic photothermal therapy; PR—plasmon resonance; PVP—polyvinylpyrrolidone; RT—radiotherapy; SWIR—short-wave infrared; TEM—transmission electron microscopy; TEOS—tetraethylorthosilicate; Vis-NIR—visible-near-infrared.
